# Bio‐functional hydrogel with antibacterial and anti‐inflammatory dual properties to combat with burn wound infection

**DOI:** 10.1002/btm2.10373

**Published:** 2022-07-05

**Authors:** Yahui Xiong, Yingbin Xu, Fei Zhou, Yanke Hu, Jingling Zhao, Zhonghua Liu, Qiyi Zhai, Shaohai Qi, Zhaoqiang Zhang, Lei Chen

**Affiliations:** ^1^ Department of Burns, Laboratory of General Surgery The First Affiliated Hospital, SunYat‐Sen University Guangzhou China; ^2^ Guangdong Provincial Engineering Technology Research Center of Burn and Wound Accurate Diagnosis and Treatment Key Technology and Series of Products SunYat‐Sen University Guangzhou China; ^3^ Institute of Precision Medicine The First Affiliated Hospital, SunYat‐Sen University Guangzhou China; ^4^ South China Agricultural University Guangzhou China; ^5^ ZhuJiang Hospital Southern Medical University Guangzhou China; ^6^ Department of Oral and Maxillofacial Surgery Stomatological Hospital, Southern Medical University Guangzhou China

**Keywords:** Ag‐metal–organic framework, burn wound infection, epigallocatechin gallate, macrophage polarization, methacrylate gelatin

## Abstract

Burn infection delays wound healing and increases the burn patient mortality. Consequently, a new dressing with antibacterial and anti‐inflammatory dual properties is urgently required for wound healing. In this study, we propose a combination of methacrylate gelatin (GelMA) hydrogel system with silver nanoparticles embed in γ‐cyclodextrin metal–organic frameworks (Ag@MOF) and hyaluronic acid‐epigallocatechin gallate (HA‐E) for the burn wound infection treatment. Ag@MOF is used as an antibacterial agent and epigallocatechin gallate (EGCG) has exhibited biological properties of anti‐inflammation and antibacterial. The GelMA/HA‐E/Ag@MOF hydrogel enjoys suitable physical properties and sustained release of Ag^+^. Meanwhile, the hydrogel has excellent biocompatibility and could promote macrophage polarization from M1 to M2. In vivo wound healing evaluations further demonstrate that the GelMA/HA‐E/Ag@MOF hydrogel reduces the number of the bacterium efficiently, accelerates wound healing, promotes early angiogenesis, and regulates immune reaction. A further evaluation indicates that the noncanonical Wnt signal pathway is significantly activated in the GelMA/HA‐E/Ag@MOF hydrogel treated group. In conclusion, the GelMA/HA‐E/Ag@MOF hydrogel could serve as a promising multifunctional dressing for the burn wound healing.

## 1 INTRODUCTION

Burn injury causes estimated 265,000 deaths every year,^1^ and burn wound infection would lead to the increasing mortality of the hospitalized burn patients. Because of the complex microenvironment, burn wounds are colonized with bacteria, which secrete much exudate and delay wound healing^2^, ^3^ compared to other forms of trauma. Besides, the inflammation from the polarization of the macrophages also plays a critical role, which could kill potential pathogens by contributing to the necessary inflammation, and when the pathogens are once cleared, the inflammation will be resolved and then the tissue remodeling and regeneration will be initiated.^4^ Currently, artificial dermal substitutes and dressing management are the main therapeutic methods for the burn wound excision.^5^ However, current commercial antibacterial dressing lacks appropriate bioactivity to regulate the complex microenvironment of the burn wound, such as the polarization of the macrophages. Therefore, new antibacterial materials have great clinical value to satisfy the aforementioned demands.^6^

The use frequency of the traditional antibiotics has decreased owing to the continuous emergency of the multidrug‐resistant bacteria. Silver nanoparticles (Ag‐NPs) are extensively used as antibacterial drugs in cutaneous wound repair^7^ because of the good antibacterial activity and little vulnerability to the bacterial resistance. However, a silver cations (Ag^+^) burst of the Ag‐NPs may increase the cytotoxicity.^8^, ^9^ In addition, small Ag‐NPs have a deficiency in stability because of the cluster aggregations. Cyclodextrin metal–organic framework (CD‐MOF) is a framework constructed by an organic ligand, cyclodextrin, with high porosity and a large specific surface area. Therefore, Ag‐NPs embedded in metal–organic frameworks (Ag@MOF) can make Ag‐NPs keep stable and be released gradually, which is an effective treatment for diabetic wounds.^10^ Prior study reveals that a hydrogel, as a drug delivery system, could allow various biomaterials to realize a controlled and sustainable release.^11^ Gelatin methacryloyl (GelMA) has the same biological properties as the natural extracellular matrix (ECM), which enhance the cell spread and proliferation.^6^, ^12^ Therefore, GelMA hydrogel could realize the sustained release of Ag@MOF.^13^

Infections in burn wounds may contribute to a local inflammatory response, an angiogenesis defect and an up‐regulated expression of the pro‐inflammation cytokines. Epigallocatechin gallate (EGCG) exerts numerous biological functions,^14^, ^15^ such as antibacterial activity, free‐radicals scavenger, regulation of the inflammation, wound healing and skin regeneration,^16^ as the major catechin in green tea. Moreover, EGCG could reduce the expression of the pro‐inflammatory factors in lipopolysaccharide (LPS)‐induced macrophages in vitro and elevate the anti‐inflammatory cytokine IL‐4,^17^ which indicates that EGCG could possibly induce the polarization of macrophages toward an anti‐inflammation phenotype. However, owing to the 1,2,3‐trihydroxyphenyl moieties in B and D ring,^18^ EGCG is susceptible to the oxidation.^19^, ^20^ The hyaluronic acids (HA) are known to have anti‐oxidant activity,^21^ and hence conjugating the HA onto EGCG to form HA‐E polymer is a feasible approach, which could reduce the rate of oxidation and remain the biological effects of EGCG^18^ by maintaining these sites during the chemical reactions.

**SCHEME 1:**
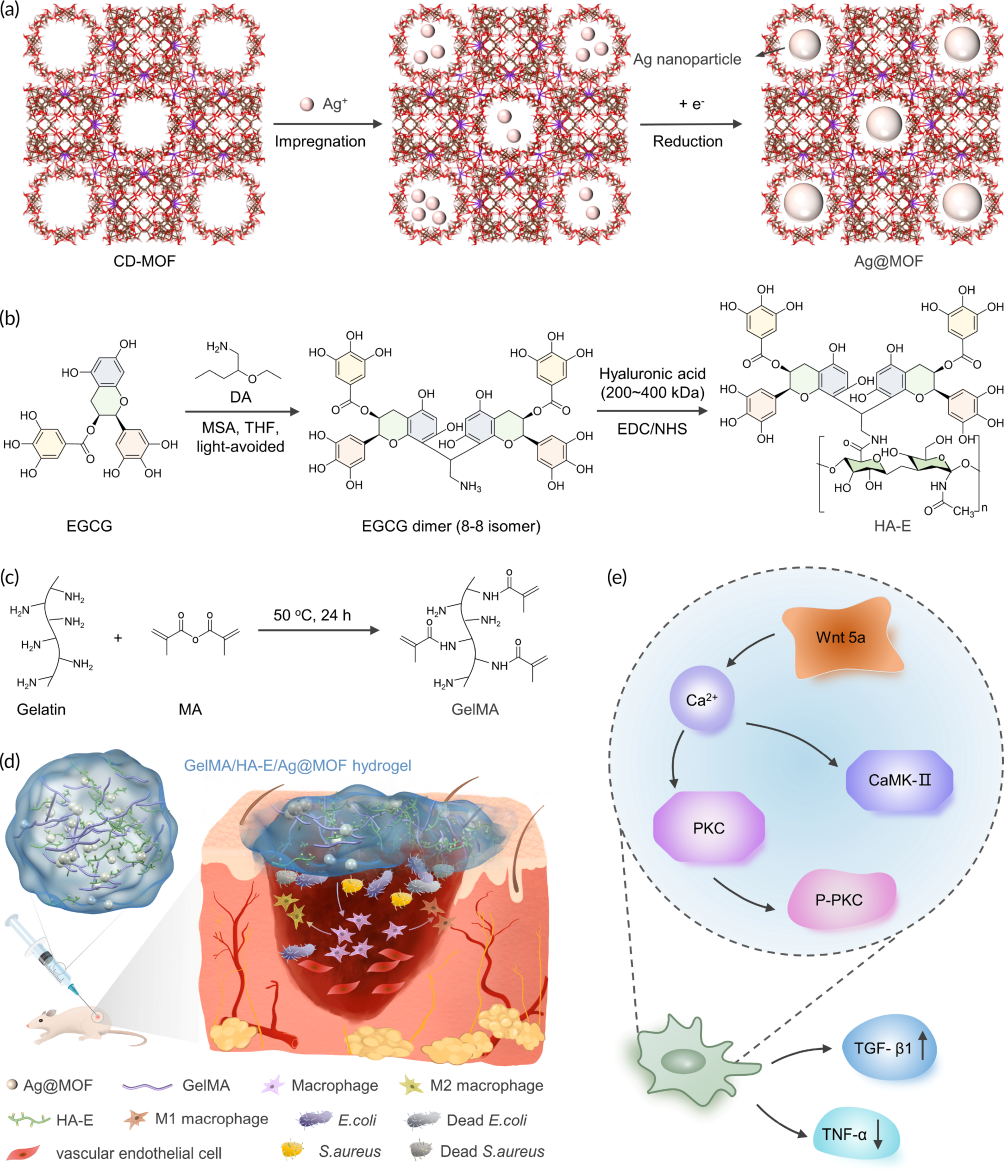
(a) Schematic of CD‐MOF template guided synthesis of Ag@MOF. (b) Schematic diagram of synthesis of HA‐E. (c) Schematic of synthesis of GelMA. (d) Schematic of infectious burn wound healing process including bacterium invasion, macrophages polarization and pro‐inflammation cytokines release. (e) Schematic diagram of activated noncanonical Wnt pathway in GelMA/HA‐E/Ag@MOF hydrogel group

To satisfy all the desired properties of a hydrogel for burn wound infection healing, we design a hydrogel system to minimize the bacterial infections at the wound (through the use of Ag@MOF), promote vascularization, regulate inflammatory (through the use of HA‐E), and finally accelerate the healing of wounds (Scheme 1). The polyphenolic groups of EGCG are reacted with aldehydes of 2‐diethoxyethylamine (DA) by Baeyer acid to form EGCG dimer, and then the tyramines of EGCG dimers are grafted to the carboxyl groups on HA by EDC/NHS chemistry. Then the physical properties, the cell compatibility and the antibacterial and anti‐inflammatory effects of the hydrogel are employed in vitro.^22^ Next, we further investigate the antibacterial, anti‐inflammation and wound healing effects in full‐thickness burn infection. Finally, the potential mechanism of the prepared hydrogels in facilitating burn wound healing is investigated for the further study.

## 2 EXPERIMENTAL SECTION

### 2.1 Synthesis of Ag@MOF

The CD‐MOF crystals were synthesized according to the previously published methods.^23^ Briefly, 97.3 g γ‐CD (biotech grade, Macklin, China) and 33.6 g KOH (90%, Macklin) were dissolved in 3 L of deionized water (DIW). After being filtrated by 0.45 μm filtration, 1.8 L of methanol (analytical grade, Sinopharm Chemical Company, China) was evaporated at 50°C for 20 min and the methanol vapor was gradually diffused into DIW. In order to trigger the crystallization, after adding 38.4 g of polyethylene glycol (PEG) (Mw = 20,000, Macklin) and a further 10 min stirring, the obtained mixture was placed overnight at ambient temperature. Finally, these CD‐MOF crystals were rinsed with anhydrous ethanol and dried overnight to collect the crystals.

The Ag@MOF was synthesized as the previously established protocols.^24^ Briefly, 600 mg of CD‐MOF crystals was dissolved in 1.5 ml of acetonitrile (analytical grade, Sinopharm Chemical Reagent Company, China), and then 5 ml AgNO_3_ precursor (20 mmol/L, Sinopharm Chemical Reagent Company) was added. The solution was centrifuged after a 72 h's reaction, and the residue was rinsed by acetonitrile for several times, and then lyophilized for further use.

### 2.2 Synthesis of GelMA and HA‐E

Depending on the reported protocols, GelMA was synthesized with slight modifications.^25^ First, 20 g of gelatin (Sigma Aldrich Corporation, USA) was fully dissolved in 250 ml DIW at 60°C. Then methacrylic anhydride (94%, Macklin) was added in the solution drop by drop at a volume of 0.6 ml/(gram gelatin). The solution was transferred into a dialysis bag, which was sank in DIW for 3–5 days at room temperature (MwCO = 8000) after reacting at ambient temperature for 8 h. Ultimately, to obtain GelMA, the collected liquid was centrifuged, filtrated by neural filter and lyophilized.

HA‐E was synthesized by following a previous report.^26^ Briefly, in a glass vial, 145 μl of 2, 2‐diethoxyethylamine (DA, Macklin) was first added in 1.2 ml of the mixture solution containing cold methane sulfonic acid (MSA, Sinopharm Chemical Reagent Company) and tetrahydrofuran (THF, Sinopharm Chemical Reagent Co., Ltd., China) (v:v = 1:5). Second, EGCG (2.29 g, Macklin) was dissolved in 1.7 μl MSA and 3.8 ml THF in advance and then dropwise added into the DA solution. After stirring at ambient temperature overnight in the dark, the liquid was evaporated until dryness. Then, the obtained solid was completely dissolved in 10 ml of DIW, which was further extracted with ethyl acetate (10 ml for five times) using a separating funnel. Next, HA (molecular weight 20 kDa, Shanghai Yuanye Biotechnology Company) and reacted EGCG dimers were used to form EGCG conjugated hyaluronic acids (HA‐E) through EDC/NHS (Shanghai Yuanye Biotechnology Company) chemistry as previously described.^27^

### 2.3 Preparation of composite hydrogels

In order to prepare the hydrogel, GelMA was fixed to 10 wt% in DIW and then adding various concentrations of HA‐E (1, 1.5, and 2 wt%) in the solution. Then, LAP (0.1 wt%, Yinchang New Material Co., Ltd., China) was added as a photoinitiator. The final mixture was added into a 48‐well plate, and then exposed to UV light (365 nm) for 10 s at the power density of 10 mW/cm^2^. For preparation of GelMA/HA‐E/Ag@MOF hydrogel, Ag@MOF was dispersed in GelMA (10 wt%) solutions with different concentrations of 20, 40, and 80 μg/ml, respectively. Then, then adding HA‐E (1 wt%) solution to prepare the mixed solutions. The prepared GelMA/HA‐E/Ag@MOF(10), GelMA/HA‐E/Ag@MOF(20) and GelMA/HA‐E/Ag@MOF(40) hydrogels were also in accordance with the above procedure.

### 2.4 Characterization of the synthesized materials

The morphological structures of Ag@MOF nanoparticles were imaged by Transmission electron micrograph (TEM; H800, Hitachi, Japan) at 200 kV. The elemental distributions of Ag@MOF were measured by energy‐dispersive x‐ray spectroscopy (EDS). The size distributions of Ag@MOF were detected and then precisely analyzed by dynamic light scattering (DLS; Zeta‐Sizer Nano‐ZS, Malvern, UK). The chemical structure of Gel, GelMA, HA, and HA‐E was characterized by ^1^H NMR (Inova‐500M, Varian, America). Fourier‐transform infrared spectroscopy (FTIR; Vertex‐70, Bruker, Germany) was utilized to determine the functional groups of Gel, GelMA, HA, and HA‐E. Scanning electron microscope (SEM; S‐3400, Hitachi, Japan) was used to obtain and analyze images of microscopic morphology of the composite hydrogels with an acceleration potential of 5 kV. Finally, the average pore size of the synthesized material was calculated by Nano measure software.

### 2.5 Physical evaluation of the composite hydrogels

#### 2.5.1 Swelling ratio of the hydrogels

According to a gravimetric method previously reported, the swelling ratio of the hydrogels was tested and then measured.^24^, ^28^ Briefly, the hydrogel samples were weighed, which was recorded as W_0_ and then completely sank into phosphate‐buffered saline (PBS, GIBCO, USA) at 37°C. The soaked hydrogels were removed from the PBS, the moisture on the hydrogel was gently absorbed by filter paper, and the swollen weight (W_t_) was noted down immediately at given time intervals (1 h, 2, 3, 4, 5 and 6 h). The following equation was used for the calculation of the swelling ratios of the hydrogels:

$$\text{Swelling ratio}=\frac{W_{t}-W_{0}}{W_{0}}\times 100\%$$

#### 2.5.2 Rheological assessment

The rheological behavior of the synthesized hydrogels (10% GelMA, 10% GelMA/1%HA‐E, 10% GelMA/1.5% HA‐E, and 10% GelMA/2%HA‐E) was tested by a TA rheometer instrument (Kinexus, Malvern Instrument, Britain) with stainless steel parallel plate rotor (25 mm). For determining the linear viscoelastic region of the hydrogels, conducting dynamic strain scanning from 0.1 to 10 rad/s^−1^ in ambient conditions and the storage modulus (*G*') and loss modulus (*G*") were obtained and then carefully measured.

#### 2.5.3 Compression test

The obtained stress/strain curve was measured through a TA rheometer instrument (AR 1500Ex; TA Instrument, USA) to extract the compressive modulus of each hydrogel. In the test, the hydrogels were placed between two compression plates and were compressed using a flat probe at 0.05 mm/s to 60%.

#### 2.5.4 Degradation in vitro

The enzymatic degradation experiments were used to measure the biodegradation performance of hydrogels. Briefly, the hydrogel samples were soaked in PBS consisting of 0 or 100 U/ml hyaluronidase, respectively, at 37°C at 70 rpm. At certain time intervals, the hydrogels were taken out, followed by deliberate procedure including washing, freeze‐drying and weighing. To illustrate the biodegradability, the degradation ratio was computed with the following equation:

$$\text{Weight loss rate}=\frac{W_{0}-W_{t}}{W_{0}}\times 100\%$$

where *W*_0_ is the original dry weight after lyophilization, and *W*_*t*_ is the dry weight after lyophilization at a designed time.

#### 2.5.5 Release of Ag^+^

The ICP‐MS method was used to detect the release of Ag^+^ from GelMA/HA‐E/Ag@MOF hydrogels. Briefly, GelMA/HA‐E/Ag@MOF hydrogels (600 μl) was added to 10 ml of PBS. At certain time (1, 2, 3, 4, 5, and 7 days), all PBS supernatant liquor was collected and then the same amount of fresh PBS were replenished instead. Lastly, the collected samples were digested with nitric acid to determine the concentrations of Ag^+^ ions.

### 2.6 In vitro antibacterial performance

*Escherichia coli* (ATCC8739), *Pseudomonas aeruginosa* (CMCCB 10104), and *Staphylococcus aureus* (ATCC‐14458) were utilized to evaluate the antibacterial activity of the presynthesized hydrogels. Briefly, the hydrogel sample (600 μl) was added in 24‐well plates and 1.8 ml mixed bacterial suspension at a final concentration of 1 × 10^8^ CFU/ml was mixed together. Following co‐culturing at 37°C for 1 day, the mixture was diluted serially, and 100 μl of each dilution was cultured at 37°C on LB agar plates. The following equation was used for the calculation of the antibacterial ratio (AR) of the hydrogels:

$$\mathrm{AR}=\frac{N\text{control}-N\text{sample}}{N\text{control}}\times 100\%$$

where *N*_control_ is the number of colonies in GelMA hydrogel and the *N*_sample_ is the number of colonies in GelMA/HA‐E or GelMA/HA‐E/Ag@MOF hydrogel.

### 2.7 In vitro biocompatibility test

#### 2.7.1 Biocompatibility test

The Cell Counting Kit‐8 assay was utilized to ascertain the cell viability. The 500 μl of GelMA, GelMA/HA‐E, and GelMA/HA‐E/Ag@MOF hydrogels was coated into 12‐well plates and then irradiated under a UV light (365 nm) for 10 s. Next, a thin layer of the prepared hydrogel was plated as a surface in the plates before 2 ml of 3 T3 cells (2 × 10^4^ cells/ml) were seeded and cultured in the medium consisting of DMEM high‐glucose medium (CGIBO, USA) with 10% fetal bovine serum (FBS, GIBCO, USA) and 1% penicillin/streptomycin at 37°C in 5% CO_2_ atmosphere. At given time (first, second, and third days), the culture medium was taken out from the plates which later added a mixed medium with CCK‐8 solution (2% [v/v]， GIBCO). After incubation for 1 h at 37°C, each well was analyzed at 450 nm (Thermo Scientific).

#### 2.7.2 Live/Dead staining

Live/Dead cell imaging kit (Kaiji Biological Technology Development Co. Ltd., China) was utilized to stain the cells to distinguish the live cells from the dead cells. Briefly, the hydrogels loaded with cells were rinsed for three times, and then 100 μl of stock solution (2 μM calcein, AM, 8 μM propidium iodide [PI]) was added to each well. Stained samples were photographed by fluorescence microscopic (Olympus FV3000; Tokyo, Japan) after the incubation at room temperature for 20 min.

#### 2.7.3 Cytoskeleton staining

TRITC phalloidin (Biyuntian Biotechnology Co., Ltd., China) and 4, 6‐diamidino‐2‐phenylindole, known as DAPI (Biyuntian Biotechnology Company, China), were used as a fluorescent dye to stain filamentous actin (F‐actin) and nuclei. In brief, the cells were rinsed with PBS (1 min for three times) followed by immersed in 4% paraformaldehyde for 30 min to fixed cells. Then, the cells were immersed in 0.5% (v:v) Triton X‐100 to increase the membrane permeability for 10 min. After rinsed for three times, the cells were stained with TRITC phalloidin for 30 min before the nuclei was stained with DAPI for 10 min in dark at room temperature. Lastly, the cell morphology of hydrogels was observed by fluorescence microscope (Olympus FV3000).

#### 2.7.4 Cell adhesion ability

For cell adhesion assay, each hydrogel samples co‐cultured with cells were washed by PBS (2 min for twice) which later was fixed in 4% paraformaldehyde for another 4 h. Replacing the liquid by PBS (2 min for twice) to wash the samples. Next, samples were dehydrated with a designed series of alcohol (30%, 50%, 75%, 95%, and 100% ethanol for 5 min each). SEM was used to observe the morphology of cells cultured on hydrogels after dried at 45°C.

### 2.8 In vitro macrophage polarization

#### 2.8.1 Induce the polarization of macrophage

RAW 264.7 macrophages were cultured in 6‐ and 24‐well plates (Corning, NY, USA) in medium‐containing DMEM media supplemented with 10% FBS. To detect the macrophage polarization induced by hydrogels, we coated the hydrogels in 6‐ and 24‐well Trans‐well chambers (Corning; polycarbonate membrane pore size: 0.4 μm) and co‐cultured with the cells for an additional 24 h in fresh DMEM, fresh DMEM containing 100 ng/ml LPS and fresh DMEM containing 10 ng/ml IL‐4 at 37°C with 5% CO_2_, respectively.

#### 2.8.2 Western Blot analysis

The proteins from the cultured RAW 264.7 cells were extracted and lysed by RIPA lysis buffer (MIK, China) with a further reaction on ice for 30 min. The total concentration of the protein collected from the cells was calculated by BCA protein assay kit (Solarbio, Beijing, China). Approximately, 10–20 μg of protein were added and then using 10% SDSPAGE gels to separate the protein, after transferring to the PVDF membrane (Millipore, Billerica, USA), in order to blocking nonspecific binding proteins, membranes were immersed in TBST containing 5% bovine serum albumin (BSA, Vetec, Brazil) for 1 h at room temperature. The primary antibodies (GAPDH [GB11002, Servicebio, 1:10,000], CD206 [DF4149; Affinity, 1:1000], and iNOS [18985‐I‐AP; Proeintech, 1: 1000]) were incubated at 4°C overnight. Secondary antibody labeled with HRP (Abcam, ab205718, 1:10,000) was used to detect the antibody‐reactive protein by incubating for 1 h at 37°C before washing in TBST for three times. Chemiluminescence western blotting detection solution (Bio‐Rad) were used to detect the protein signal on the Alpha Innotech Fluor Chem imaging system after rinsing in TBST for three times.

#### 2.8.3 RT‐PCR analysis

Total RNA in the cultured RAW 264.7 cells was isolated and purified by RNA‐Quick Purification Kit (RN001; Shanghai Yishan Biotechnology CO., Ltd) as described in its instruction. Using PrimeScript™ RT reagent Kit with gDNA Eraser (RR047A; Takara Biomedical technology, Beijing, China) to synthesize the cDNAs. The RT‐PCR was performed on QuantStudio™ 1 Real‐Time PCR System using SYBR™ Green Master Mix (A25742, Thermofish) according to the manufacturer's instruction. Primer sequences were listed in Table S1. Results were normalized to GAPDH mRNA. All samples were assayed in triplicate.

### 2.9 In vivo wound healing evaluation

#### 2.9.1 Full‐thickness skin burn model

The animal work was reviewed and approved by the Animal Ethics Committee of South China Agricultural University (SYXK [Guangdong] 2019‐0136) and maintained under standard conditions. Male Sprague–Dawley (SD) rats weighing 200–250 g were purchased from Guangdong Medical Laboratory Animal Center. After fed and maintained for 3 days to adapt the specific pathogen‐free environment, the rats were randomly divided into five groups, three rats in each group: a gauze group, a GelMA hydrogel group, a GelMA/HA‐E hydrogel group, a GelMA/HA‐E/Ag@MOF hydrogel group, and an Aquacel Ag dressing group. Before the surgery, the dorsal hair of the rats was removed by the hair removal cream after anesthetized with pentobarbital (45 mg/kg). Four scald burn wounds (12 mm diameter) were established by attaching a cropper hammer on their back with intervals of 2 cm. After 3 h, four full‐thickness skin wounds (12 mm) were established on each back of the rat. The 40 μl of bacteria suspension, which was prepared by mixing 20 μl of *E. coli* and 20 μl of *S. aureus* at a certain concentration of 1 × 10^8^ CFU/ml was applied on each wound site. Different treatments began in 24 h after the bacterial challenge as assigned above, and the wound sites were further covered by sterile Tegaderm (3 M) dressings.

#### 2.9.2 Wound healing analysis

The appearance and the size of the wounds sites were photographed to calculate the wound areas by using IPP 6.0 software (Media Cybernetics, USA). Changes appeared at different days of wound areas were shown as remaining percentage. As designed, rats were sacrificed at certain time intervals (3rd, 7th, 10th, and 14th days) and the skin samples excised from the wound site were fixed in 4% paraformaldehyde for histological analyses. The remaining wound area percentage of each group was precisely calculated using the following equation:

$$\text{Wound area}=\frac{\text{Open area}\;\mathrm{on}\;\text{the indicated}\;\mathrm{day}\;}{\text{Original wound area}}\times 100\%$$

#### 2.9.3 In vivo antibacterial activity evaluation

In order to evaluate the bacterial growth in rat wounds, skin tissues on the Day 3 were homogenized with 2 ml of sterile normal saline, and then the homogenate was diluted. A 100 μl of homogenate was painted well on gram‐negative bacteria selection medium (*E. coli*) and mannitol salt AGAR medium plates (*S. aureus*), respectively. The plates were incubated at 37°C and cultured upside down. After 24 h culture, the bacteria on the culture medium was photographed and the number was recorded, consisting three parallel samples in each group.

### 2.10 Histology and immunohistochemistry analysis

The skin tissue samples excised from the back of the rats were fixed, embedded and cross‐sectioned to 4 μm thickness slices. To evaluate the histomorphology of the regenerative skin tissue, sections were stained routinely with HE staining for histological studies and Masson staining. To assess the inflammatory responses and the macrophage polarization condition in vivo, the slices from the tissue on the Day 7 were stained with immunohistochemical staining stained with TNF‐α (Abcam, ab6671, 1:500), TGF‐β1 (Affinity, AF1027, 1: 00), iNOS (Proeintech, 18985‐I‐AP, 1:500), CD68 (Abcam, ab53444, 1:500), CD163 (Abcam, ab182422, 1:500), and CRR7 (Abcam, ab32527, 1:500), respectively. The slides were observed and photos were taken by the inverted fluorescence microscope (IX83; Olympus, Japan).

### 2.11 Western blot of wound tissues

For western blot analysis for tissues, the skin samples excised from rats in each group were completely homogenized with proteinase‐containing protein extraction reagent for 10 times. After centrifuged at 10,000 rpm for 20 min at 4°C, the supernatant was collected to another tube so as to perform a quantitative analysis by protein assay kit. The proteins (50 μg) were loaded and heated to 95°C for 10 min followed by other experimental steps as described previously.

### 2.12 Statistical analysis

All experiments in this study performed were repeated at least three times with three wells repeated per treatment. The collected data was performed as means ± SD. Utilizing one‐way analysis variance (ANOVA) analysis by SPSS 25 (IBM). A *p* value of <0.05 was considered significant (*p < 0.05, **p < 0.01, ***p < 0.001).

## 3 RESULTS AND DISCUSSION

### 3.1 Characterization of nanoparticles and hydrogel

**FIGURE 1:**
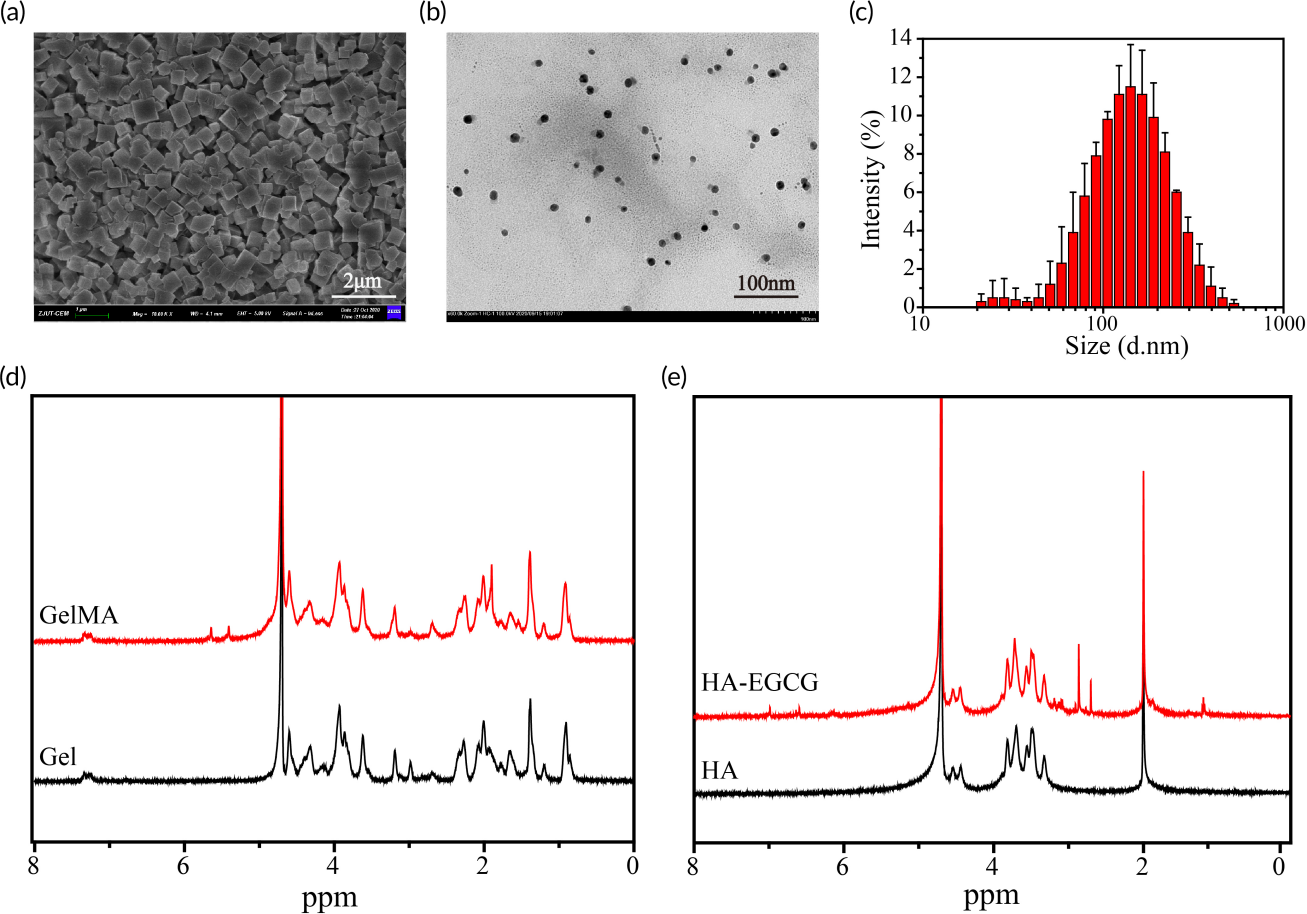
(a) A scanning electron microscope (SEM) image of the cyclodextrin metal–organic framework (CD‐MOF). (b) A TEM image of AgNPs embedded in metal‐organic frameworks (Ag@MOF). (c) The size distribution of Ag@MOF. (d) ^1^H NMR spectra of gelatin and GelMA. (e) ^1^H NMR spectra of HA and HA‐E

The SEM image demonstrated that the obtained CD‐MOF appeared as individual regular crystals and had a uniform size around 143 nm (Figure 1a), because of the organic framework linked by γ‐cyclodextrins (γ‐CDs) and alkali metal salts. The TEM image indicated that the synthesized Ag@MOF had a spherical shape with a small size (Figure 1b). The hydrodynamic size of Ag@MOF was 120 nm ± 3.392 nm with polydispersity (PDI) of 0.241 (Figure 1c). EDS spectrum displayed higher percentage of silver signals for Ag@MOF samples, which implies the formation of Ag NPs within CD‐MOF crystals (Figure S1). Prior study reported CD‐MOF accelerated the synthesis of the ultrafine Ag NPs nanoparticles since it reduced AgNO_3_ into Ag NPs and its small pore in CD‐MOF prevented further nucleation.^23^

^1^H‐NMR was used to verify the chemical structure of GelMA and HA‐E. The characteristic methacrylate peaks in GelMA shown in Figure 1d were 5.41 and 5.64 ppm, which might belong to the C=C peak in methacrylic anhydride (MA) and this result indicated that MA was successfully grafted onto gelatin.^29^ Additionally, a characteristic resonance of EGCG (δ = 6.99, 6.62, and 6.15 ppm) was observed in HA‐E (Figure 1e), assigning to the hydrogen peaks of the benzene ring in EGCG, which verified the successful preparation of HA‐E.^30^ Moreover, such chemical synthesis of HA‐E can maintain the biological function of EGCG, which mainly derived from its function groups.^18^ Further, FTIR demonstrated Gel‐, HA‐, MA‐, and EGCG‐related peaks at around 1200 cm^−1^ (Figure S2), indicating that our results were consistent with the previous references [31]. Figure S3A showed the cross‐linking network hydrogel was formed through mixing the GelMA solution and the HA‐E solution under UV‐light irradiation.^32^ Figure S3B displayed the image appearance of the GelMA/HA‐E hydrogels with various HA‐E concentrations.

**FIGURE 2:**
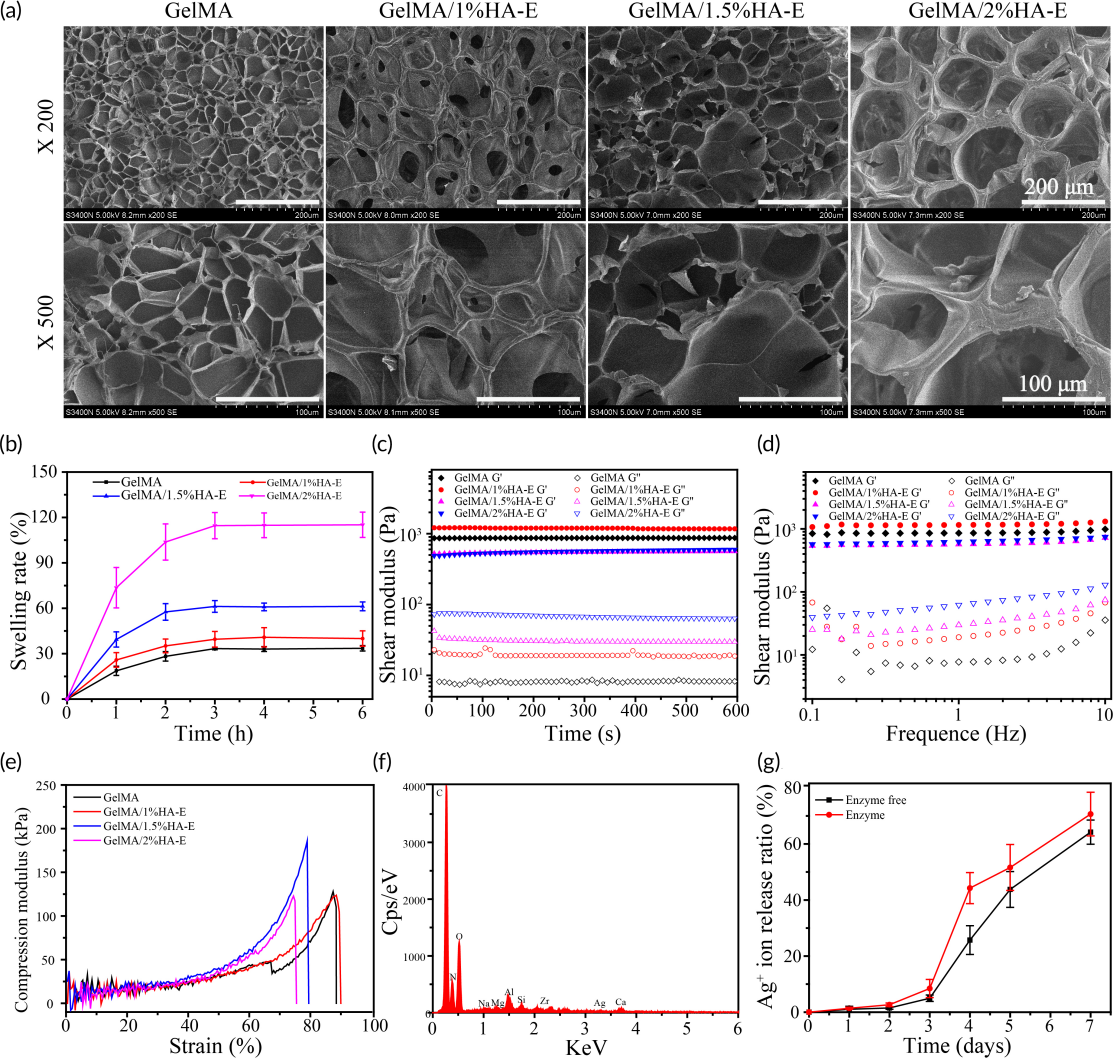
(a) Scanning electron microscope (SEM) images of GelMA, GelMA/1% HA‐E, GelMA/1.5% HA‐E, GelMA/2% HA‐E hydrogels. (b) The swelling ratio. (c) The rheological properties. (d) The storage modulus and the loss modulus vary with frequency ranging from 0.1 to 10 Hz. (e) The compression–strain curves of the hydrogels. (f) The energy‐dispersive x‐ray spectroscopy (EDS) spectra of GelMA/HA‐E/Ag@MOF. (g) The cumulative release curve of Ag^+^ in GelMA/HA‐E/Ag@MOF hydrogel

The porous structure and the pore size of all hydrogels were observed by SEM after the freeze‐drying. The SEM images of hydrogels demonstrated an irregular and porous network structure (Figure 2a), which could facilitate the transmission of the oxygen and the nutrition, absorb the exudate and maintain the suitable moisture environment in wound healing.^33^ Meanwhile, the average pore size of GelMA, GelMA/1%HA‐E, GelMA/1.5%HA‐E, and GelMA/2%HA‐E hydrogels were 31.1 ± 14.1, 59.9 ± 18.4, 61.5 ± 16.6, and 108.7 ± 29.5 μm, respectively (Figure S4). With an increase in the HA‐E concentration, the average pore size grew. The GelMA/1% HA‐E had an appropriate pore size for cell migration and differentiation,^34^ since a medium pore diameter ranging from 54 to 70 μm rarely impact the cell behavior and in vivo reaction as previously reported.^35^

### 3.2 Swelling ratio analysis

The water absorption properties of the hydrogels could be indicated by the swelling results. The pore size has an impact on the swelling degree of the hydrogel,^36^ which finally affects its mechanical strength. With the increasing soaking time, the swelling rate of all hydrogels increased rapidly and reached the maximum and the swelling equilibrium within 3 h (Figure 2b). The swelling ratio of the GelMA/1%HA‐E hydrogel (39.5% ± 5.1%) was well below the hydrogel loaded with 1.5% and 2% HA‐E (61.2% ± 3.9% and 114.5% ± 8.6%), which was slightly higher than that of GelMA hydrogel (33.3% ± 0.8%). These results indicated that the addition of 1% HA‐E may maintained the excellent structural stability without notable swelling.

### 3.3 Rheological and compression properties

Referring to the previous study, the viscoelastic properties of the hydrogels were used to assay the stability of the cross‐linked networks.^37^ Figure 2c demonstrated that the storage modulus (*G*') of all hydrogels were significantly larger than the loss modulus (*G*''), which implied their solid elastic attribute. *G*'' of GelMA/HA‐E hydrogel grew with the increase of HA‐E concentration, indicating that there was a higher cross‐linking construction in the mixture hydrogel system. Meanwhile, *G*' of GelMA/1%HA‐E was two orders of magnitude higher than *G*'' (Figure 2d), which indicated that the GelMA/1%HA‐E hydrogel had the predominant elastic response among all HA‐E‐loaded hydrogels.

The appropriate mechanical properties of the hydrogels are of major significance in numerous biomedical applications. The compression modulus of GelMA/1.5%HA‐E was the highest, up to 175 kPa at 80%, showing strong rigidity (Figure 2e). Meanwhile, the compression modulus of GelMA/1%HA‐E was 125 kPa, peaking at nearly 90%, which demonstrated the hydrogel could withstand a large deformation without being damaged under a certain compression owing to the high crosslinking density and complex structure. In addition, the curves indicated that all hydrogels could maintain their shapes after being compressed up to 70%, which were greater than the dermis of the human skin. In our study, it is demonstrated that through adding HA‐E, the compression property of our synthesized hydrogels be increased and GelMA/1%HA‐E hydrogel exhibit outstanding tenacity property.

### 3.4 Ag^+^ release studies and in vitro degradation

The EDS spectrum showed that Ag was coexisted with other elements (Figure 2f), which demonstrated that Ag‐NPs were homogeneously distributed in the hydrogels. The ICP‐MS was used to evaluate whether the prepared hydrogels had the property of slowing down the release of Ag^+^. As shown in Figure 2g, GelMA/HA‐E/Ag@MOF hydrogel exhibited a sustainable release of the Ag^+^ in the first 3 days. However, a rapid release growth of Ag^+^ occurred at the fourth day, which indicated that it could maintain a high concentration of Ag^+^ in the early stage of the infection wound. Until the Day 7, the Ag^+^ release ratio of the hydrogel was 64.2% ± 4.3% and 70.7% ± 7.8% in solution containing either lysozyme or not, respectively. The slow and long‐lasting release of Ag^+^ in the early stage was due to that Ag nanoparticles existed in the form of elemental Ag. The conversion of Ag^+^ was depending on the hydrolysis of elemental Ag. The rapid release of Ag^+^ in the late stage may be due to that Ag nanoparticles participate in the oxidation of HA‐E. All results demonstrated that the GelMA/HA‐E/Ag@MOF hydrogel had good sustained‐release effect for Ag^+^.

The weight loss curves of the hydrogels showed a gradual decrease in Figure S5A, but there was a sharp dropping rate in lysozyme group (Figure S5B). Compared with pure GelMA hydrogel (44.4% ± 1.4%, 65.3% ± 2.1%) at the fifth day in the presence and absence of lysozyme, the degradation rate was relatively sharp as the increasing of the concentration of HA‐E, ranging from 49.9% ± 2.0% to 65.7% ± 1.6% and 67.2% ± 1.6% to 84.3% ± 1.7% respectively.^38^ Both GelMA and HA‐E have the sequence to react with lysozyme.^38^, ^39^ When the hydrogel is degraded, it is advantageous for the loaded material to be released from the hydrogel in a sustainable speed and fully cleared from it to accumulate in the intended site.^40^ Hence, the appropriate degradation rate could not only maintain the stability of the hydrogel but also promote the drug consistently release.

### 3.5 In vitro antibacterial evaluation

**FIGURE 3:**
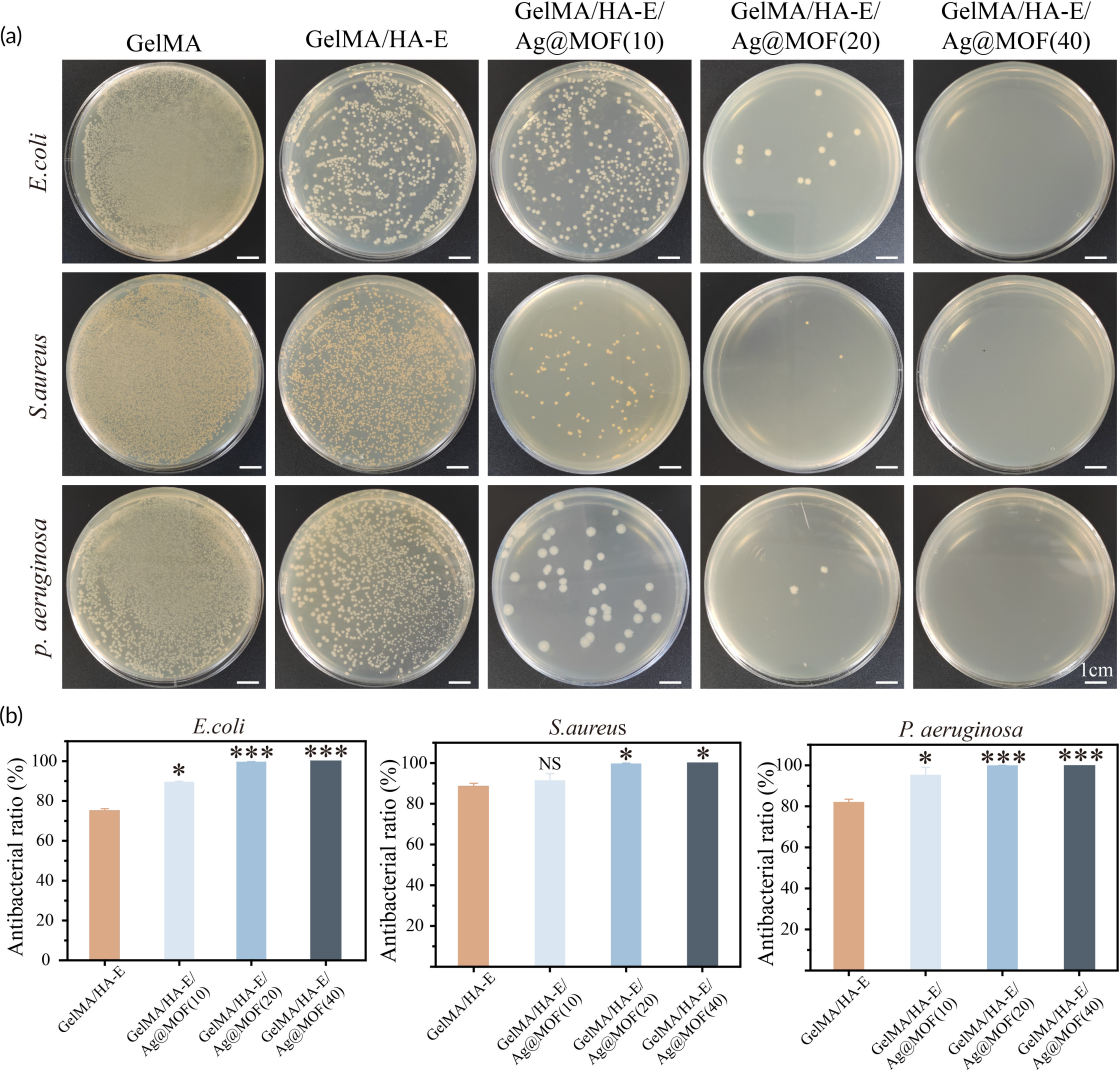
(a) Bacterial colony counts of the hydrogels toward *E. coli*, *S. aureus*, and *P. aeruginosa*. (b) Quantitative analysis of antibacterial ratio (*p < 0.05, **p < 0.01, ***p < 0.005)

*S. aureus*, *E. coli*, and *P. aeruginosa* were used as representative pathogens in skin infection to evaluate the antibacterial properties of the hydrogels in vitro. After the co‐incubation with GelMA, GelMA/HA‐E and GelMA/HA‐E/Ag@MOF (Ag@MOF from 0 to 40 μg/ml) at 37°C in vitro for 24 h, the results of the antibacterial activity are presented in Figure 3a,b. Compared with the pure GelMA hydrogel, with the adding of HA‐E, the numbers of bacterial colony‐forming units (CFU) were reduced to 75.3% ± 0.8% for *E. coli*, 88.8% ± 1.3% for *S. aureus*, and 82.1% ± 1.4% for *P. aeruginosa*. In addition, all hydrogels loaded with Ag‐NPs (GelMA/HA‐E/Ag@MOF) exhibited much better antibacterial property with the antibacterial ratio of >95%, which increased to 100% when Ag@MOF was 40 μg/ml. Prior studies reported that the Ag‐NPs and EGCG had antibacterial property, which were verified by the results.^41^ Considering the biocompatibility and the antibacterial ratio, 20 μg/ml of Ag@MOF was selected for the following experiment.

### 3.6 Biocompatibility of the hydrogels

**FIGURE 4:**
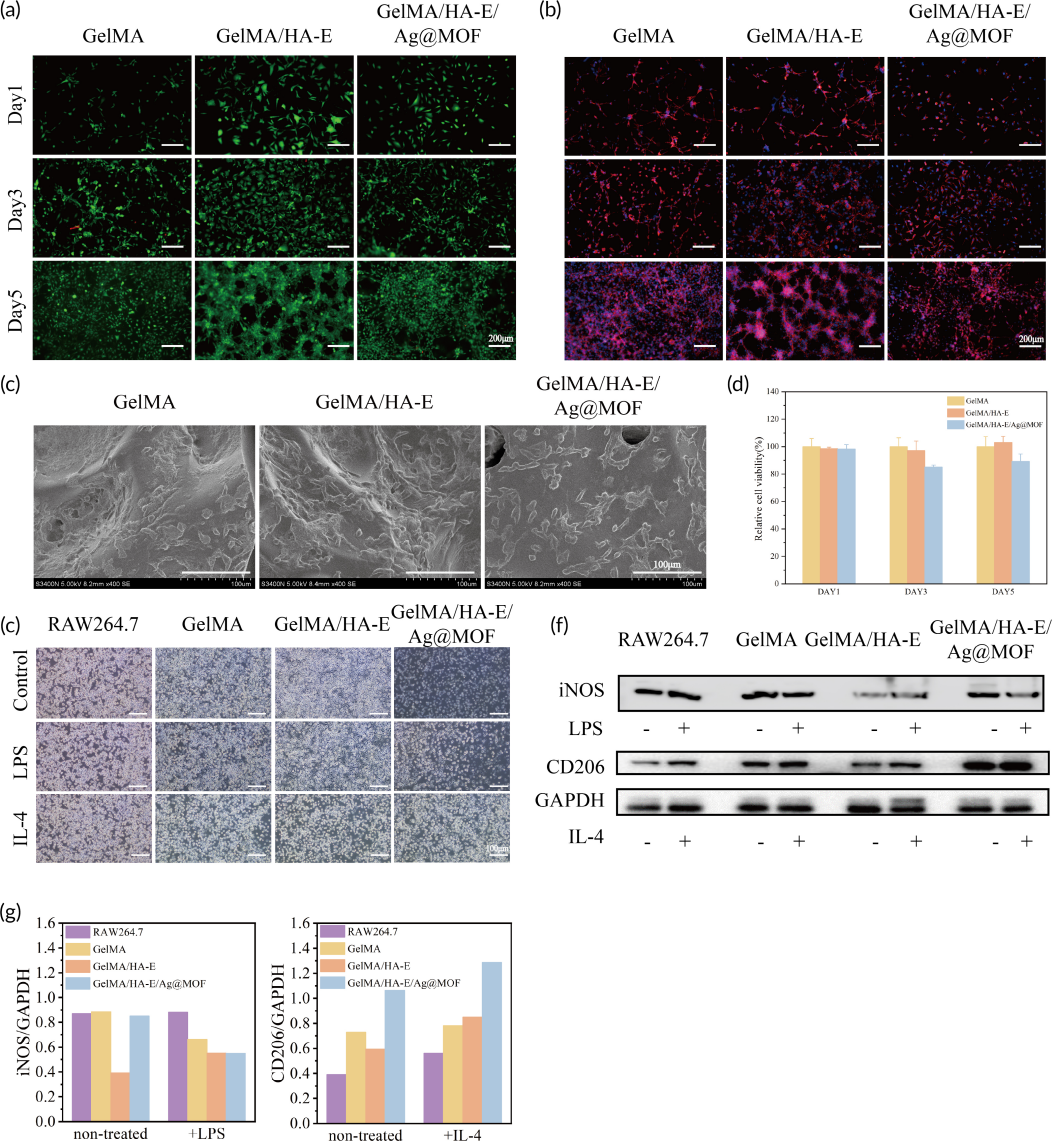
(a) Representative live/dead staining images of 3T3 cells cultured on GelMA, GelMA/HA‐E, and GelMA/HA‐E/Ag@MOF hydrogels. (b) Cytoskeleton staining images of 3T3 cells. (c) SEM images of 3T3 cells cultured on the hydrogels. (d) Cell viability of 3T3 cells cultured on the hydrogels by CCK‐8 assay. (e) Images of M1 and M2 macrophages in RAW 264.7 cells. (f) Western blot analysis of macrophages polarization. (g) Quantitative analysis of WB results

The biocompatibility of the prepared hydrogels was assessed using 3 T3 cells through CCK‐8, live/dead staining and F‐actin staining.^36^ The live/dead staining results revealed higher density of the viable cells in the GelMA/HA‐E hydrogel group than those in the other two groups with few dead cells (red) present and normal cell morphology (Figure 4a). Resembling the prior live/dead staining results, the cytoskeleton architecture of 3T3 cells in all groups showed that the cells were spindle‐shaped morphology during the experiment (Figure 4b). Moreover, GelMA/HA‐E group showed a number of cell–cell contacts at the fifth day, which formed an interconnected network. These results revealed that the hydrogel surface could promote the cells to elongate and bond tightly with porous structure to supply nutrition and assisted in proliferation.

Next, the morphology of 3T3 cells, which were in advanced cultured on the surface of the prepared hydrogels were observed at the given time. From the SEM images (Figure 4c), it was demonstrated that 3T3 cells were well attached on the surface of all hydrogels, which implied that the hydrogel was nontoxic and had good cell biocompatibility and could support cell attachment, migration, and proliferation. As shown in Figure 4d, the cell viability of GelMA/HA‐E hydrogel group were both over 95% at the first day and the third day, which indicated the good cytocompatibility of all hydrogels. Meanwhile, the cell viability of GelMA/HA‐E/Ag@MOF hydrogel groups decreased from 98.3% ± 3.2% to 85.1% ± 1.4%, which indicated the Ag@MOF were gradually released from the hydrogels and had impact on the cells. At the fifth day, the cell viability of the GelMA/HA‐E/Ag@MOF hydrogel group was 89.3% ± 5.4%, which meant that the Ag^+^ release from GelMA/HA‐E/Ag@MOF hydrogel reached a balance and the hydrogel had a good biocompatibility. All results confirmed that GelMA/HA‐E/Ag@MOF hydrogel had excellent biocompatibility and was well suited for such biomaterial applications.

### 3.7 In vitro polarization of macrophages

Macrophages were first classified into M1 macrophages (a proinflammatory phenotype) and M2 macrophages (an anti‐inflammatory phenotype).^42^ The hydrogel with the ability to facilitate the M1‐to‐M2 transition of the macrophages would be favorable for the wound healing. In Figure 4e, the macrophages under the treatment displayed an elongated and irregular morphology. In addition, the in vitro modulatory effect of the GelMA/HA‐E/Ag@MOF hydrogel on the macrophages polarization was evaluated by Western blot (WB) and RT‐PCR. For further detecting the surface makers, the untreated macrophages, and the expression of iNOS (M1 marker) and CD206 (M2 marker) were determined as shown in Figure 4f,g. The cells on the GelMA/HA‐E/Ag@MOF hydrogel displayed significantly higher expression levels of CD206 than the cells co‐cultured did with pure GelMA and GelMA/HA‐E hydrogels. In contrast, the expression of iNOS in the GelMA/HA‐E/Ag@MOF hydrogel was relatively lower than those in the other groups. The Ag^+^ released from GelMA/HA‐E/Ag@MOF hydrogel played a key role in causing M1 macrophage apoptosis and ROS scavenging, resulting in repolarizing M1 macrophages to M2 macrophages.^43^ The RT‐PCR results (Figure S6) showed that GelMA/HA‐E/Ag@MOF hydrogel remarkably reduced the ratio between the expression of TNF‐α and CD206 in IL‐4‐treated macrophages compared with the LPS treated group in vitro. These results suggested the GelMA/HA‐E/Ag@MOF hydrogel have the strongest ability to facilitate the polarization of RAW 264.7 macrophages.

### 3.8 In vivo evaluation of wound healing effect in infection burn wound

#### 3.8.1 Wound healing examination

**FIGURE 5:**
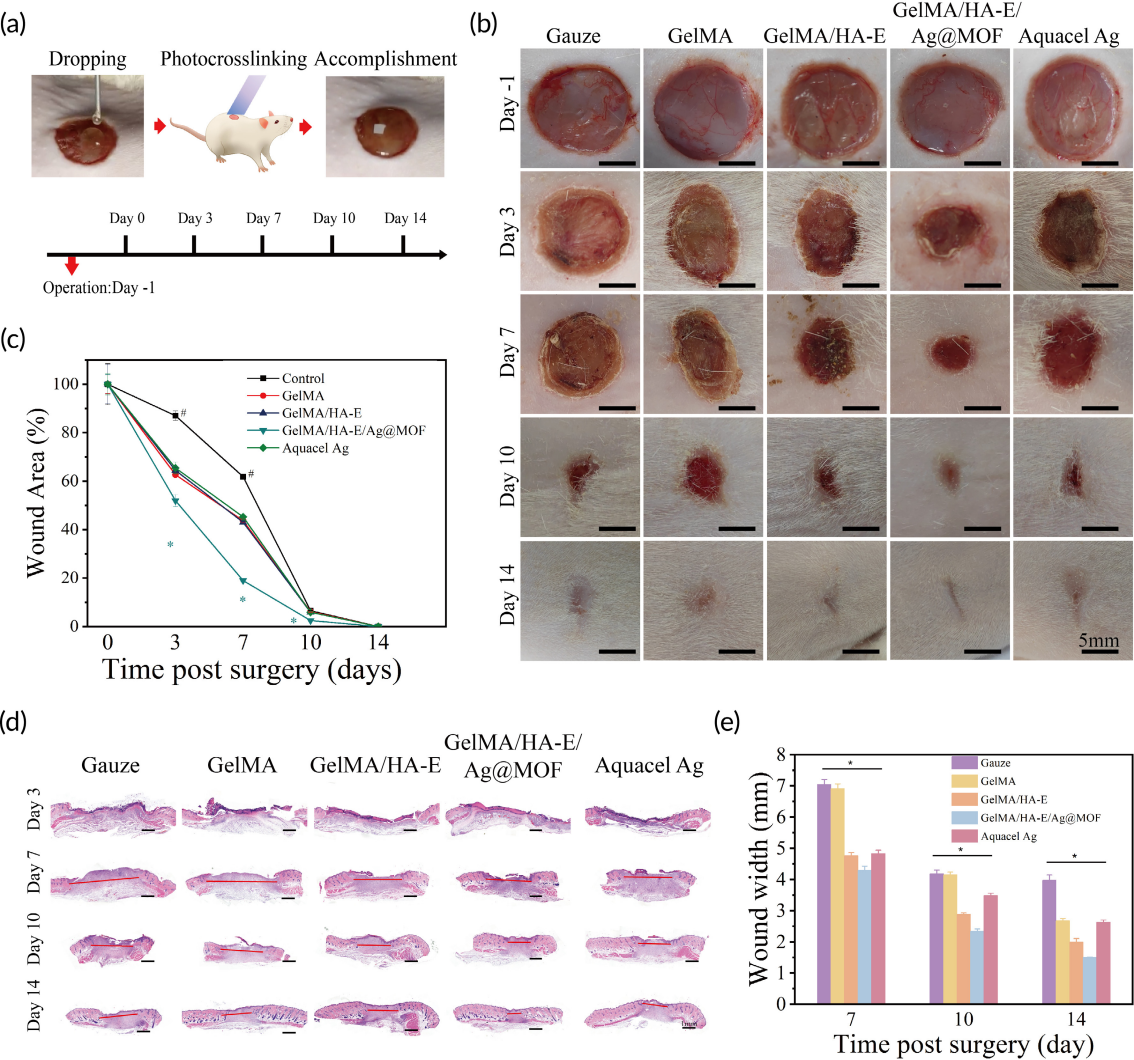
(a) The process of the infectious burn wound model. (b) Photographs of the wounds treated with gauze, GelMA, GelMA/HA‐E, GelMA/HA‐E/Ag@MOF and Aquacel Ag at the 3rd, 7th, 10th, and 14th days. (c) Quantitative analysis of wound size at the 3rd, 7th, 10th, and 14th days (*p < 0.05, between the GelMA/HA‐E/Ag@MOF and others, #*p* < 0.05, between gauze group and others). (d) Representative images of wound section gap from the Day 3 to the Day 14 (indicated by red lines). (e) Quantitative analysis of wound section gap from the Day 7 to the Day 14 (*p < 0.05 between groups, except gauze vs. GelMA and GelMA/HA‐E vs. Aquacel Ag at the 7th day, gauze vs. GelMA at the 10th day and GelMA vs. Aquacel Ag at the 14th day)

The above results prove that GelMA/HA‐E/Ag@MOF hydrogel with the desired physical and antibacterial properties may have a potential for the treatment of the burn wound infection. Besides the infection, wound healing in burn injuries are accompanied with a large number of biochemical reactions, inflammation regulating, and vascular skin deficits rebuilding, which could be strongly improved by the hydrogel dressing. In this study, GelMA, GelMA/HA‐E, GelMA/HA‐E/Ag@MOF hydrogel, and Aquacel Ag were employed to evaluate the treatment for the burn wound infection. Figure 5a displayed the process of injecting the hydrogels on the infectious burn wound, which was the same injectable as previous study.^7^ As shown in Figure 5b,c, wound area at the Day 0 was recorded and measured as initial area (100%). Compared with others group (86.99% ± 1.9%, 62.66% ± 0.7%, 64.47% ± 1.2%, and 65.47% ± 2.4%), the wound area (%) in GelMA/HA‐E/Ag@MOF group (51.98% ± 2.5%) was significantly smaller and healed better at the third day after the surgery. When this tendency continued until the seventh day, between GelMA/HA‐E/Ag@MOF group (18.95% ± 0.6%) and the gauze group (61.87% ± 0.8%), it was shown a significant difference in wound size. At the 10th day, GelMA/HA‐E/Ag@MOF group showed the smallest wound area (2.47% ± 0.2%) among the groups, which was basically healed, while the other groups with approximate 6% left. At the 14th day, there were no obvious wound could be seen in all groups, which indicated the epidermis was almost closed.

To further explore the therapeutic efficacy of the hydrogel on the regeneration of epidermis and dermis from a microscopic point of view, histological analysis such as hematoxylin and eosin (HE) and Masson staining were performed.^44^ The closure of the wound was measured by calculating the gap of the wound in HE staining images (Figure 5d). Since the wound tissues were difficult to calculate at the third day, Figure 5e displayed the tendency of the wound from the 7th day to the 14th day post the surgery. Although the gap of the wound gradually grew small with time, the gap of the wound in GelMA/HA‐E/Ag@MOF hydrogel group was the smallest at the seventh day (4.29 ± 0.14 mm), which displayed a significant difference compared with other groups (7.04 ± 0.16, 6.91 ± 0.15, 4.76 ± 0.10, and 4.83 ± 0.11 mm). In view of the significant difference between the gauze group and the GelMA/HA‐E/Ag@MOF hydrogel, they were involved in the following histological analysis.

#### 3.8.2 In vivo antibacterial efficiency evaluation

Wound infections are one of the most common complication in burn injury,^45^ because of the weak resistance to the organisms originated from the patients' own skin and the contact with the environments. Given that GelMA/HA‐E/Ag@MOF hydrogel possessed powerful antibacterial property as proved, we then explored whether it could exhibit the same antimicrobial property against microorganisms in vivo. The tissues applied mixed bacteria suspension (*S. aureus* and *E. coli*) were collected from the wound tissue to investigate the bacterial counts of the infected burn wounds at the third day. Afterward, the corresponding CFU on the dish was photographed so that the antibacterial ability of GelMA/HA‐E/Ag@MOF hydrogels could be evaluated.

**FIGURE 6:**
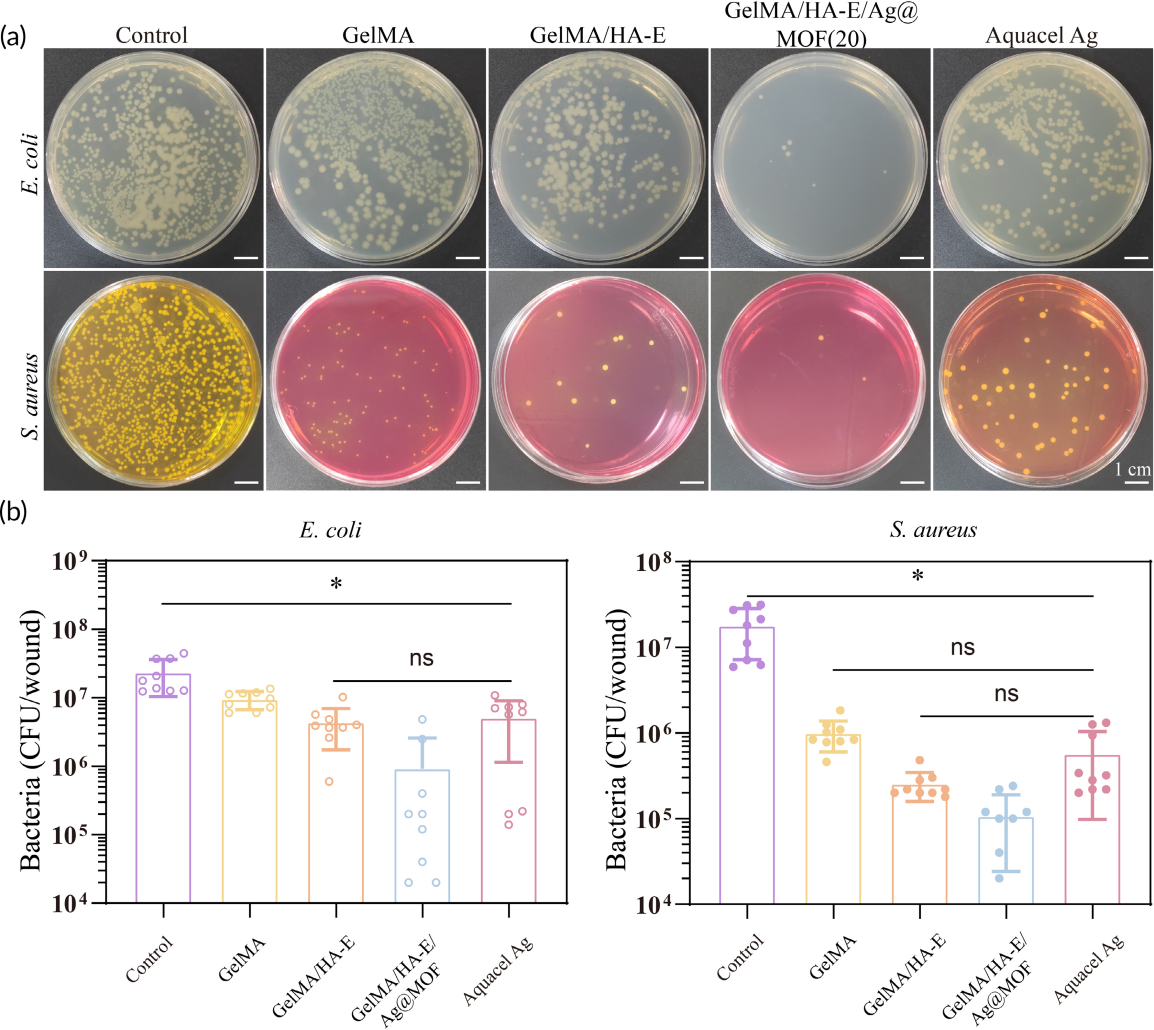
(a) Photographs of *E. coli* and *S. aureus* colonies obtained from the wound tissue. (b) Quantitative analysis the number of bacteria (*p < 0.05)

The prepared GelMA/HA‐E/Ag@MOF hydrogel exhibited strong antibacterial ability toward *E. coli* and *S. aureus* (Figure 6a). At the same time, in GelMA/HA‐E/Ag@MOF group, the number of the bacterial colonies was significantly reduced.

The results demonstrated that the number of the bacteria in the wound treated by the gauze was about 2.3 × 10^7^ CFU/wound for *E. coli* and 1.8 × 10^7^ CFU/wound for *S. aureus*, GelMA group was about 9.4 × 10^6^ CFU/wound for *E. coli* and 9.9 × 10^5^ CFU/wound for *S. aureus*, GelMA/HA‐E group was about 4.4 × 10^6^ CFU/wound for *E. coli* and 2.5 × 10^5^ CFU/wound for *S. aureus* (Figure 6b). However, the antibacterial efficiency of GelMA/HA‐E/Ag@MOF (*E. coli* was 9.2 × 10^5^ CFU/wound, *S. aureus* was 1.1 × 10^5^ CFU/wound) was better than that of the positive control group, Aquacel Ag group (*E. coli* was 5.1 × 10^6^ CFU/wound, *S. aureus* was 5.7 × 10^5^ CFU/wound). These results revealed that the GelMA/HA‐E showed more efficient antibacterial property against gram‐positive bacteria than the gram‐negative ones. Whereas GelMA/HA‐E/Ag@MOF hydrogels had the best synergistically antibacterial ability against both the gram‐negative and the gram‐positive organisms.

#### 3.8.3 Histological analysis

The wound healing process can be subdivided into four processes: hemostasis, inflammation, proliferation, and remodulation.^46^ The inflammatory phase serves to prevent infection and activate signals required for the wound repair. Besides the infection, the burn wound also suffers from the uncontrolled immune response and hyperexuberant cytokine production during the inflammatory phase (including hemostasis and inflammation).^46^ In the proliferative phase, a new blood vessel‐network was constructed to supply the newly formed granulation tissue, which could bring oxygen and nutrients to the wound bed.^47^

**FIGURE 7:**
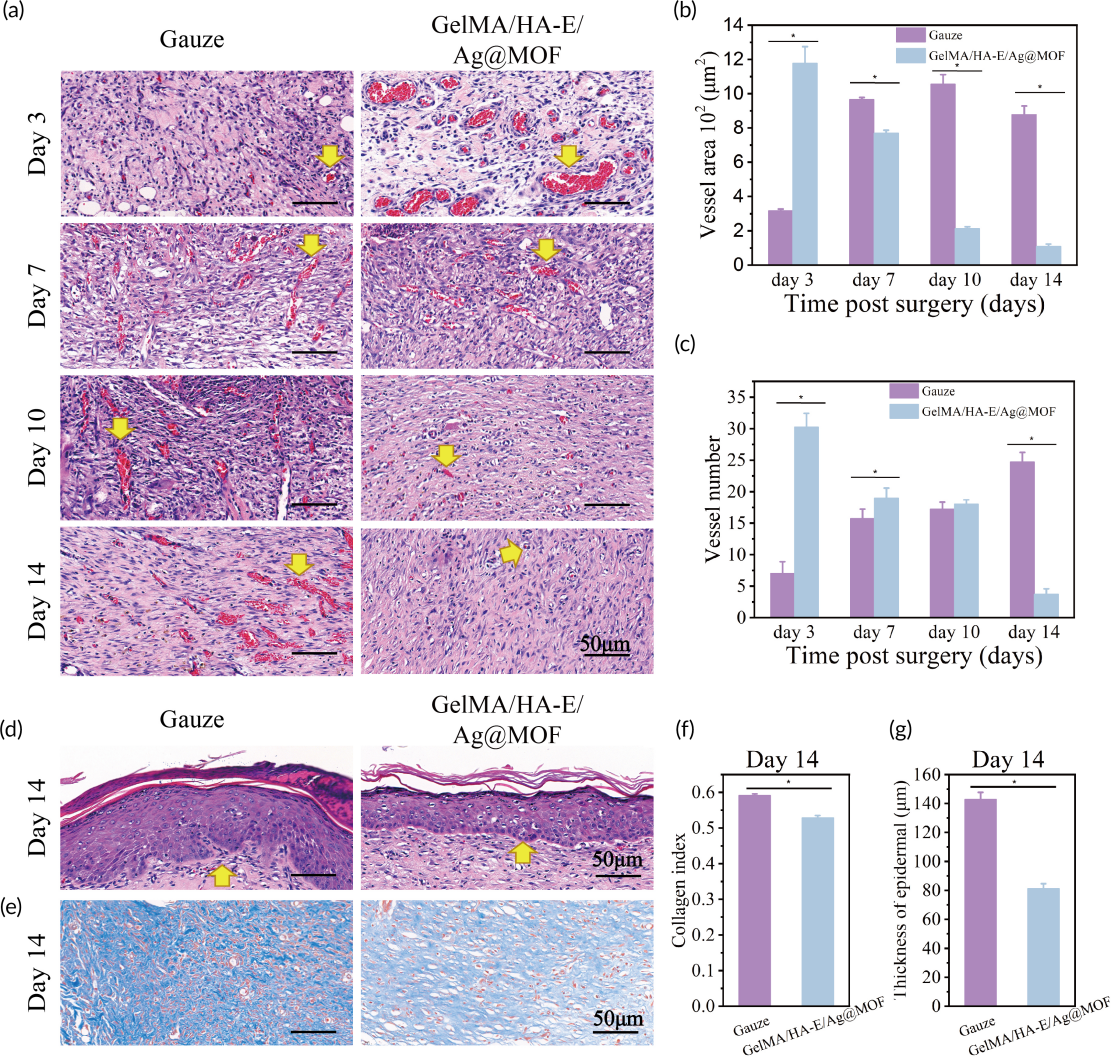
(a) Representative images of vessels in wound sections by HE staining from the Day 3 to the Day 14 (indicated by yellow arrows). (b,c) Quantitative analysis of vessel area and vessels number in wounds sections from the 3rd day to the 14th day (*p < 0.05 between groups, except GelMA/HA‐E vs Aquacel Ag at the 3rd day and the 14th day). (d) Representative images of dermal in wound sections by HE staining (indicated by yellow arrows). (e) Representative images of collagen deposition in wound sections by Masson staining. (f) Quantitative analysis of the epidermal thickness in wounds sections at the 14th day (*p < 0.05). (g) Quantitative analysis of the collagen deposition in wounds sections at the 14th day (*p < 0.05)

Severe burn wounds lose much dermal blood flow and hence neovascularization plays a key role in the wound healing.^48^ The HE staining was used to assay the morphology and area of the microvessels in the wound section. The microvessels were clearly seen in all wound with the presence of blood cells inside the lumen, which illustrated the establishment of an efficacious blood circulation to supply nutrients and remove waste.^49^ At the third day (Figure 7a,b), the microvessel area in the gauze group (317.43 ± 9.1 μm^2^) was significantly lower than that in GelMA/HA‐E/Ag@MOF hydrogel group (1178.74 ± 95.99 μm^2^), which indicated that GelMA/HA‐E/Ag@MOF hydrogel had efficient proangiogenic capacity at the early stage of the healing process. While the area of the vessels significantly increased in the control group up to the 14th day (877.93 ± 50.75 μm^2^), and the area of the vessels in GelMA/HA‐E/Ag@MOF hydrogel group decreased to 109.91 ± 12.99 μm^2^. The small area of the vessels could be due to that the late stage of the wound healing does not need the formation of the blood vessels. Figure 7c also displayed the same tendency: the number of the microvessels of GelMA/HA‐E/Ag@MOF hydrogel group gradually decreased while that of the gauze group increased. Structurally, gelatin in the GelMA also contains integrin‐binding motif (RGD) and matrix metallopeptidases (MMPs) sequences, which could stimulate the angiogenesis.^39^ In addition, this result (Figure S7) verified that HA‐E could also promote early neovascularization in wound through maintaining their bio‐function.^50^ The early neovascularization could provide nutrition for epithelial regeneration and facilitate wound healing. In contrast, the area of the microvessels in the control group increased during the late phase of wound healing, attributing to the infection and the inflammation. Hence, the results demonstrated that with the antibacterial activity from Ag^+^ in early stage of wound healing process, the GelMA/HA‐E/Ag@MOF may be more efficient for promoting wound neovascularization.^7^

Further, the epithelial cells resurface the injury and the epidermis was rebuilt in maturation or remodeling phase. The thickness of the epidermal in GelMA/HA‐E/Ag@MOF hydrogel group (81.24 ± 3.32 μm) was as good as the normal skin with massive appendage structures of the skin in the dermis,^49^ while the epidermis thickness of the control group was much higher than all the other groups at the 14th day (Figure 7d,f). The new granulation tissue was composed by the collagen and the ECM during the proliferation phase. Collagen is important components for the skin structure and function reconstitution. At the 14th day, mature collagen (light blue and loosely packed) fibers were present in GelMA/HA‐E/Ag@MOF hydrogel, while the collagen in the control group was still in an admixed dysplastic state (Figure 7e). The quantitative analysis results further supported these observations. The collagen deposited in GelMA/HA‐E/Ag@MOF was significantly fewer than that in the gauze groups (Figure 7g). These observations revealed that the synthesized hydrogel could induce sufficient early vascularization, promote epidermis regeneration, and mitigate wound fibrosis so as to help the lesion site damage close to the normal skin.

### 3.9 In vivo effect of hydrogel on macrophages polarization and inflammation microenvironment

Macrophages play a crucial role in various physiological and pathological processes in early wound infections.^51^ As widely distributed innate immune cells, pro‐inflammatory phenotype macrophages effectively defend against pathogens and promote wound healing. Following successful resuscitation, burn wound then face the chronic inflammation, which may impair the wound healing.^47^ Macrophages mainly display a pro‐inflammatory phenotype (M1‐like) and produce a wide array of the inflammatory cytokines (i.e., TNF‐α), which induce tissue destruction and healing delay. Besides, the inflammation phase in wounds should transition into the proliferation phase in the following steps. Macrophage infiltration is vital step in the wound healing process.^52^ When the number of M2 phenotype macrophages is significantly increased, which will produce high expressions of anti‐inflammatory cytokines (i.e., TGF‐β1) to reduce the macrophages infiltration, accelerate tissue repair, and generate a favorable anti‐inflammatory microenvironment.^53^ If macrophages could not successfully prompt macrophages to shift toward the two phenotype, which will increase secretion of foreign body giant cell (FBGC) formation and fibrous enhancer factor, resulting in the healing delay.^54^

**FIGURE 8:**
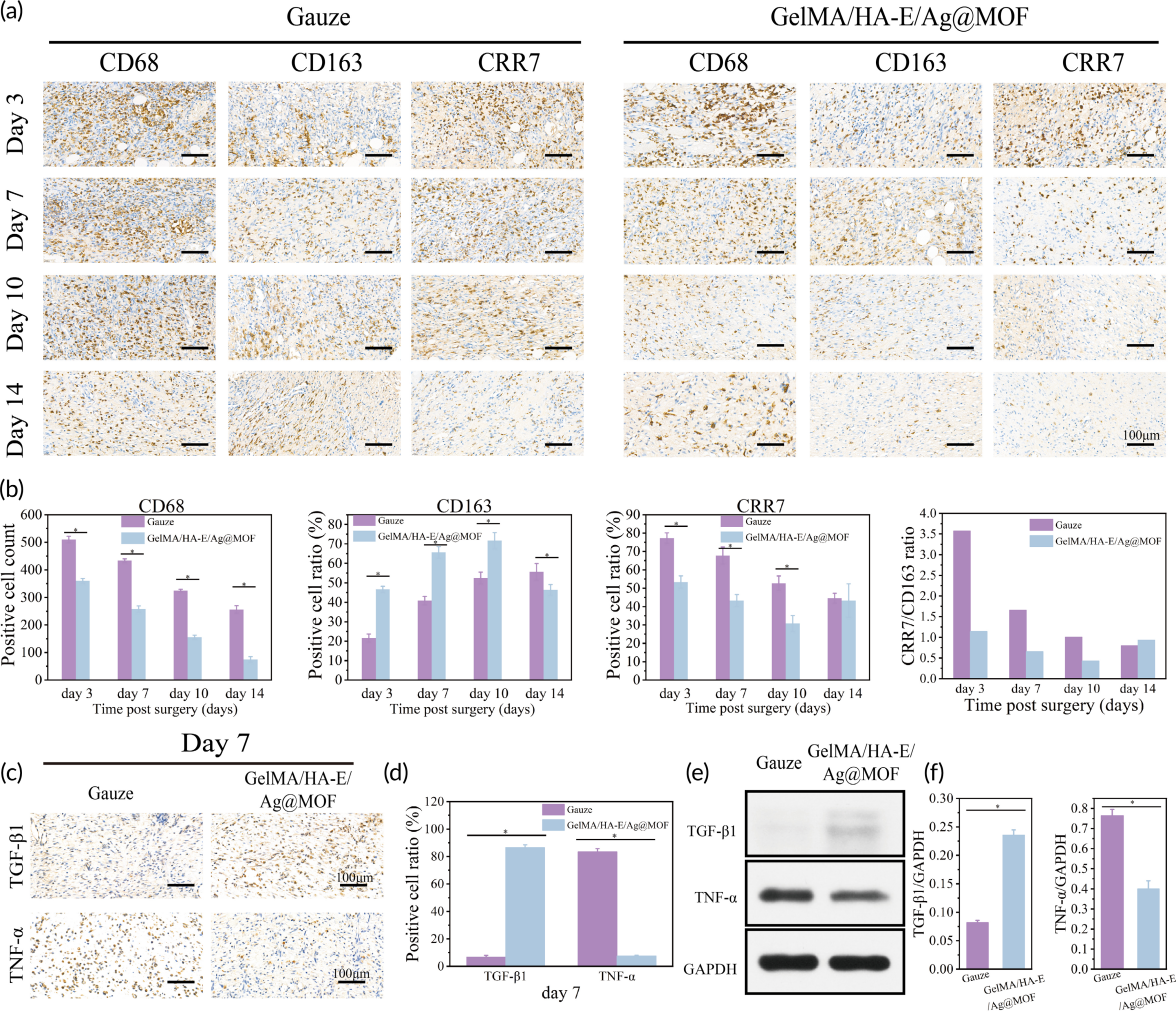
(a) Representative images of CD68, CD63, and CRR7 expression at wounds by IHC staining. (b) Quantitative analysis of CD68, CD63, and CRR7 expression. (c) Representative images of TGF‐β1 and TNF‐α expression at wounds by IHC staining. (d) Quantitative analysis of TGF‐β1 and TNF‐α expression. (e) Western bolt analysis of TGF‐β1 and TNF‐α. (f) Quantitative analysis of TGF‐β1 and TNF‐α

As previously proved in vitro, GelMA/HA‐E/Ag@MOF hydrogel could facilitate M2 polarization of the macrophages, we inferred that whether the material could also play an inflammation regulating role in vivo. To characterize the phenotype of the macrophage cells at the seventh day, we stained the tissue cells with CD68, CRR7 (M1 marker), and CD163 (M2 marker) antibodies by immunohistochemistry staining. As shown in Figure 8a,b, the inflammation cells around the wound sites were less in GelMA/HA‐E/Ag@MOF group than those in the gauze treated group, which displayed less CRR7‐positive cells and more CD163‐positive cells. It intuitively demonstrated that the GelMA/HA‐E/Ag@MOF hydrogels cause more macrophages to be polarized to M2 phenotype in vivo. At different time point, there were significant difference in proportion of M1/M2 between the gauze group and GelMA/HA‐E/Ag@MOF hydrogel (*P* < 0.05). It was remarkable that the ratio of M1/M2 was decreased over time in both groups. In addition, compared to the gauze group, the ratio of M1/M2 in GelMA/HA‐E/Ag@MOF hydrogel group was obvious low at the third day, which indicated that the prepared hydrogel could polarize macrophages toward the M2 phenotype at the early stage of healing.

To further investigate the mechanisms of GelMA/HA‐E/Ag@MOF hydrogel on accelerating wound healing and reducing inflammatory response, the wound tissues were obtained at the seventh day after the surgery and the corresponding expressions of chemokines were first detected by immunohistochemical and western bolt analysis assays (Figure 8c–f). These results indicated that GelMA/HA‐E/Ag@MOF hydrogel group displayed more anti‐inflammatory cytokine (TGF‐β1) expression than the gauze group. Contrarily, the expression of pro‐inflammatory cytokine (TNF‐α) significantly decreased in GelMA/HA‐E/Ag@MOF hydrogel group compared to gauze group. All results revealed GelMA/HA‐E/Ag@MOF hydrogel could polarize macrophages to M2 phenotype of the wounds, which might consequently recruit cells and modulate their proliferation and differentiation to provide an immune‐regulating environment for favor wound healing.

### 3.10 Noncanonical Wnt signaling pathway

**FIGURE 9:**
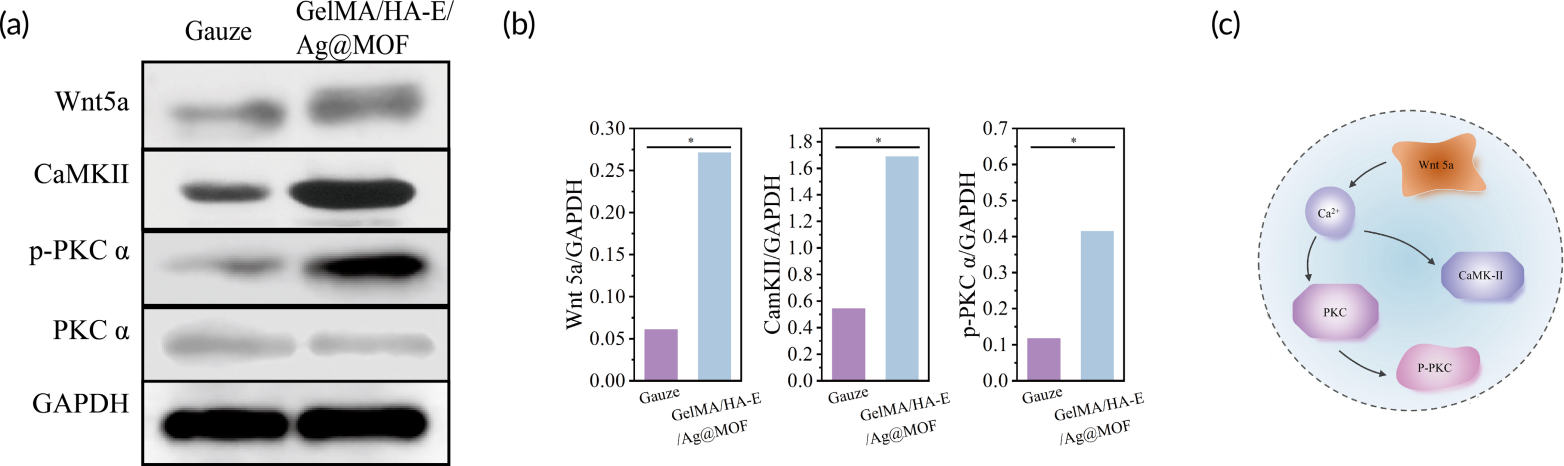
(a) Western bolt analysis of Wnt5a, CamKII, p‐PKC α, and PKC α. (b) Quantitative analysis of Wnt5a, CamKII, and p‐PKC α (*p < 0.05). (c) Schematic diagram of activated noncanonical Wnt pathway

Having established that GelMA/HA‐E/Ag@MOF hydrogel preferentially polarized macrophages toward an M2 phenotype, we sought to examine the signaling events involved in this transition. Prior studies reported that the noncanonical components of the Wnt pathway were correlated with the wound infection, which had attracted great attention in recent years.^55^, ^56^ The marker of noncanonical Wnt signal pathway (Wnt 5a) could be detected at the early stage of inflammation caused by implantation,^57^ and the downstream such as CamKII/PKC suppresses canonical Wnt signaling, which aids the expression of TGF‐β.^58^ Wnt5a‐mediated noncanonical Wnt‐signaling could regulate the endothelial cell proliferation.^59^ Therefore, we evaluated the makers of noncanonical Wnt signal pathway and its downstream factors (Wnt 5a, CamKII, p‐PCKα, and PCKα) to explore whether the noncanonical Wnt signal pathway was activated process in the infection burn wound by the GelMA/HA‐E/Ag@MOF hydrogel. As shown in the western blot results (Figure 9a,b), at the seventh day the expressions of Wnt5a and CamKII were significantly higher in GelMA/HA‐E/Ag@MOF group than those in the control group, which indicated that the noncanonical Wnt signal pathway was activated in vivo experiment (Figure 9c). These findings suggested that noncanonical Wnt signal pathway and their products serve an activation in the wound sites of GelMA/HA‐E/Ag@MOF group.

## 4 CONCLUSIONS

In summary, we successfully prepared HA‐E and Ag@MOF‐loaded GelMA hydrogels for the treatment of burn wound infections in rats. By adjusting the concentration of HA‐E, the excellent pore‐size distribution, appropriate physical properties and antibacterial activity make GelMA/1%HA‐E/Ag@MOF suitable for wound healing application. The biocompatibility tests demonstrated that the GelMA/HA‐E/Ag@MOF (20 μg/ml) hydrogel obviously promoted the proliferation, the elongation and the interaction of 3T3 cells and the polarization of the anti‐inflammatory phenotype macrophages. Furthermore, in vivo wound healing of the burn wound infection indicated that GelMA/HA‐E/Ag@MOF hydrogel contributed to promote the wound healing through facilitating the regeneration of the epithelial wounds, protecting the wound rebuilding microvessel network, reducing the inflammation‐induced infiltration, enhancing the collagen deposition, and inducing the macrophages to the anti‐inflammatory phenotype with noncanonical Wnt signal pathway activated. Overall, our results suggested that GelMA/HA‐E/Ag@MOF hydrogel be able to become a novel candidate for wound dressing with great potential for accelerating the burn wound infection healing.

## AUTHOR CONTRIBUTIONS

**Yahui Xiong:** Data curation (equal); methodology (equal); writing – original draft (equal). **Yingbin Xu:** Conceptualization (equal); methodology (equal); writing – original draft (supporting). **Fei Zhou:** Data curation (equal); validation (equal). **Yanke Hu:** Validation (equal). **Jingling Zhao:** Data curation (equal); supervision (equal). **Zhonghua Liu:** Methodology (equal). **Qiyi Zhai:** Funding acquisition (supporting); methodology (equal). **Shaohai Qi:** Conceptualization (equal); writing – review and editing (equal). **Zhaoqiang Zhang:** Conceptualization (equal); writing – review and editing (lead). **Lei Chen:** Conceptualization (lead); funding acquisition (lead); project administration (equal); writing – review and editing (lead).

## CONFLICT OF INTERESTS

The authors declare that they have no competing interests.

## Supporting information


**Appendix S1** Supporting Information

## Data Availability

The data that support the findings of this study are available from the corresponding author upon reasonable request.

## References

[1] Mofazzal Jahromi M , Sahandi Zangabad P , Moosavi Basri S , et al. Nanomedicine and advanced technologies for burns: preventing infection and facilitating wound healing. Adv Drug Deliv Rev. 2018;123:33‐64. 28782570 10.1016/j.addr.2017.08.001 PMC5742034

[2] Zhou L , Liu N , Feng L , et al. Multifunctional electrospun asymmetric wettable membrane containing black phosphorus/Rg1 for enhancing infected wound healing. Bioeng Transl Med. 2022;7(2):e10274. 35600652 10.1002/btm2.10274 PMC9115714

[3] Yuan Y , Shen S , Fan D . A physicochemical double cross‐linked multifunctional hydrogel for dynamic burn wound healing: shape adaptability, injectable self‐healing property and enhanced adhesion. Biomaterials. 2021;276:120838. 34274780 10.1016/j.biomaterials.2021.120838

[4] Liu Y , Cui J , Wang H , et al. Enhanced therapeutic effects of MSC‐derived extracellular vesicles with an injectable collagen matrix for experimental acute kidney injury treatment. Stem Cell Res Ther. 2020;11(1):161. 32321594 10.1186/s13287-020-01668-w PMC7178991

[5] Amini‐Nik S , Yousuf Y , Jeschke M . Scar management in burn injuries using drug delivery and molecular signaling: current treatments and future directions. Adv Drug Deliv Rev. 2018;123:135‐154. 28757325 10.1016/j.addr.2017.07.017 PMC5742037

[6] He W , Bai J , Chen X , et al. Reversible dougong structured receptor‐ligand recognition for building dynamic extracellular matrix mimics. Proc Natl Acad Sci U S A. 2022;119(8):e2117221119. 10.1073/pnas.2117221119 PMC8872741 35181608

[7] Chen H , Cheng R , Zhao X , et al. An injectable self‐healing coordinative hydrogel with antibacterial and angiogenic properties for diabetic skin wound repair. NPG Asia Mater. 2019;11(1):3.

[8] Lu Z , Gao J , He Q , et al. Enhanced antibacterial and wound healing activities of microporous chitosan‐Ag/ZnO composite dressing. Carbohydr Polym. 2017;156:460‐469. 27842847 10.1016/j.carbpol.2016.09.051

[9] AshaRani P , Mun GLK , Hande M , Valiyaveettil S . Cytotoxicity and genotoxicity of silver nanoparticles in human cells. ACS Nano. 2009;3(2):279‐290. 19236062 10.1021/nn800596w

[10] Zhang X , Li G , Wu D , et al. Recent progress in the design fabrication of metal‐organic frameworks‐based nanozymes and their applications to sensing and cancer therapy. Biosens Bioelectron. 2019;137:178‐198. 31100598 10.1016/j.bios.2019.04.061

[11] Hu H , Dong L , Bu Z , et al. miR‐23a‐3p‐abundant small extracellular vesicles released from Gelma/nanoclay hydrogel for cartilage regeneration. J Extracell Vesicles. 2020;9(1):1778883. 32939233 10.1080/20013078.2020.1778883 PMC7480606

[12] Rehman S , Augustine R , Zahid A , Ahmed R , Tariq M , Hasan A . Reduced graphene oxide incorporated GelMA hydrogel promotes angiogenesis for wound healing applications. Int J Nanomed. 2019;14:9603‐9617. 10.2147/IJN.S218120 PMC6901121 31824154

[13] Nguyen K , Richards L , Massarsky A , Moon T , Tayabali A . Toxicological evaluation of representative silver nanoparticles in macrophages and epithelial cells. Toxicol Vitro. 2016;33:163‐173. 10.1016/j.tiv.2016.03.004 26975774

[14] Singh B , Shankar S , Srivastava R . Green tea catechin, epigallocatechin‐3‐gallate (EGCG): mechanisms, perspectives and clinical applications. Biochem Pharmacol. 2011;82(12):1807‐1821. 21827739 10.1016/j.bcp.2011.07.093 PMC4082721

[15] Zhong Y , Shahidi F . Lipophilized epigallocatechin gallate (EGCG) derivatives as novel antioxidants. J Agric Food Chem. 2011;59(12):6526‐6533. 21526762 10.1021/jf201050j

[16] Ma Y , Liu G , Tang M , Fang J , Jiang H . Epigallocatechin gallate can protect mice from acute stress induced by LPS while stabilizing gut microbes and serum metabolites levels. Front Immunol. 2021;12:640305. 33868268 10.3389/fimmu.2021.640305 PMC8047319

[17] Luo P , Wang F , Wong N , et al. Divergent roles of Kupffer cell TLR2/3 signaling in alcoholic liver disease and the protective role of EGCG. Cell Mol Gastroenterol Hepatol. 2020;9(1):145‐160. 31562937 10.1016/j.jcmgh.2019.09.002 PMC6909006

[18] Kim SH , Kim K , Kim BS , et al. Fabrication of polyphenol‐incorporated anti‐inflammatory hydrogel via high‐affinity enzymatic crosslinking for wet tissue adhesion. Biomaterials. 2020;242:119905. 32145505 10.1016/j.biomaterials.2020.119905

[19] Cho JH , Lee JS , Shin J , et al. Ascidian‐inspired fast‐forming hydrogel system for versatile biomedical applications: pyrogallol chemistry for dual modes of crosslinking mechanism. Adv Funct Mater. 2018;28(6):1705244.

[20] del Marmol V , Beermann F . Tyrosinase and related proteins in mammalian pigmentation. FEBS Lett. 1996;381(3):165‐168. 8601447 10.1016/0014-5793(96)00109-3

[21] Lee F , Lim J , Reithofer MR , et al. Synthesis and bioactivity of a conjugate composed of green tea catechins and hyaluronic acid11Electronic supplementary information (ESI) available: HSQC spectrum, 1H spectra and figures of radical scavenging activities. Polym Chem. 2015;6(24):4462‐4472. doi:10.1039/c5py00495k

[22] Zhao X , Pei D , Yang Y , et al. Green tea derivative driven smart hydrogels with desired functions for chronic diabetic wound treatment. Adv Funct Mater. 2021;31(18):2009442.

[23] Shakya S , He Y , Ren X , et al. Ultrafine silver nanoparticles embedded in cyclodextrin metal‐organic frameworks with GRGDS functionalization to promote antibacterial and wound healing application. Small. 2019;15(27):e1901065. 31069948 10.1002/smll.201901065

[24] Wei Y , Han S , Walker DA , Fuller PE , Grzybowski BA . Nanoparticle Core/Shell architectures within MOF crystals synthesized by reaction diffusion. Angew Chem Int Ed. 2012;51(30):7435‐7439. 10.1002/anie.201202549 22753323

[25] Zhou F , Hong Y , Liang R , et al. Rapid printing of bio‐inspired 3D tissue constructs for skin regeneration. Biomaterials. 2020;258:120287. 32847683 10.1016/j.biomaterials.2020.120287

[26] Lee F , Lim J , Reithofer MR , et al. Synthesis and bioactivity of a conjugate composed of green tea catechins and hyaluronic acid. Polym Chem. 2015;6(24):4462‐4472.

[27] Lee F , Chung JE , Xu K , Kurisawa M . Injectable degradation‐resistant hyaluronic acid hydrogels cross‐linked via the oxidative coupling of green tea Catechin. ACS Macro Lett. 2015;4(9):957‐960. 35596463 10.1021/acsmacrolett.5b00544

[28] Wang Y , Wang X , Shi J , et al. A biomimetic silk fibroin/sodium alginate composite scaffold for soft tissue engineering. Sci Rep. 2016;6(1):39477. 27996001 10.1038/srep39477 PMC5172375

[29] Zhu M , Wang Y , Ferracci G , Zheng J , Cho N , Lee B . Gelatin methacryloyl and its hydrogels with an exceptional degree of controllability and batch‐to‐batch consistency. Sci Rep. 2019;9(1):6863. 31053756 10.1038/s41598-019-42186-x PMC6499775

[30] Chen J , Yang J , Wang L , et al. Modified hyaluronic acid hydrogels with chemical groups that facilitate adhesion to host tissues enhance cartilage regeneration. Bioactive Mater. 2021;6(6):1689‐1698. 10.1016/j.bioactmat.2020.11.020 PMC7708943 33313448

[31] Yi X , He J , Wang X , et al. Tunable mechanical antibacterial, and cytocompatible hydrogels based on a functionalized dual network of metal coordination bonds and covalent crosslinking. ACS Appl Mater Interfaces. 2018;10(7):6190‐6198. 29381319 10.1021/acsami.7b18821

[32] Yue K , Trujillo‐de Santiago G , Alvarez MM , Tamayol A , Annabi N , Khademhosseini A . Synthesis, properties, and biomedical applications of gelatin methacryloyl (GelMA) hydrogels. Biomaterials. 2015;73:254‐271. 26414409 10.1016/j.biomaterials.2015.08.045 PMC4610009

[33] Lin Z , Wu T , Wang W , et al. Biofunctions of antimicrobial peptide‐conjugated alginate/hyaluronic acid/collagen wound dressings promote wound healing of a mixed‐bacteria‐infected wound. Int J Biol Macromol. 2019;140:330‐342. 31421174 10.1016/j.ijbiomac.2019.08.087

[34] Forero‐Doria O , Polo E , Marican A , et al. Supramolecular hydrogels based on cellulose for sustained release of therapeutic substances with antimicrobial and wound healing properties. Carbohydr Polym. 2020;242:116383. 32564841 10.1016/j.carbpol.2020.116383

[35] Bryant SJ , Cuy JL , Hauch KD , Ratner BD . Photo‐patterning of porous hydrogels for tissue engineering. Biomaterials. 2007;28(19):2978‐2986. 17397918 10.1016/j.biomaterials.2006.11.033 PMC1950475

[36] Yoon H , Shin S , Cha J , et al. Cold water fish gelatin methacryloyl hydrogel for tissue engineering application. PLoS One. 2016;11(10):e0163902. 27723807 10.1371/journal.pone.0163902 PMC5056724

[37] Chen F , Ni Y , Liu B , et al. Self‐crosslinking and injectable hyaluronic acid/RGD‐functionalized pectin hydrogel for cartilage tissue engineering. Carbohydr Polym. 2017;166:31‐44. 28385238 10.1016/j.carbpol.2017.02.059

[38] Liang K , Ng S , Lee F , et al. Targeted intracellular protein delivery based on hyaluronic acid–green tea catechin nanogels. Acta Biomater. 2016;33:142‐152. 26785145 10.1016/j.actbio.2016.01.011

[39] Heltmann‐Meyer S , Steiner D , Müller C , et al. Gelatin methacryloyl is a slow degrading material allowing vascularization and long‐term use in vivo. Biomed Mater. 2021;16:6. 10.1088/1748-605X/ac1e9d 34406979

[40] Ayoubi‐Joshaghani MH , Seidi K , Azizi M , et al. Potential applications of advanced Nano/hydrogels in biomedicine: static, dynamic, multi‐stage, and bioinspired. Adv Funct Mater. 2020;30(45):2004098.

[41] Ouyang J , Zhu K , Liu Z , Huang J . Prooxidant effects of epigallocatechin‐3‐gallate in health benefits and potential adverse effect. Oxid Med Cell Longev. 2020;2020:9723686. 32850004 10.1155/2020/9723686 PMC7441425

[42] Atri C , Guerfali FZ , Laouini D . Role of human macrophage polarization in inflammation during infectious diseases. Int J Mol Sci. 2018;19(6):1801. 10.3390/ijms19061801 PMC6032107 29921749

[43] Chen Y , Guan M , Ren R , et al. Improved immunoregulation of ultra‐low‐dose silver nanoparticle‐loaded TiO(2) nanotubes via M2 macrophage polarization by regulating GLUT1 and autophagy. Int J Nanomed. 2020;15:2011‐2026. 10.2147/IJN.S242919 PMC7102919 32273699

[44] Liu W , Ou‐Yang W , Zhang C , et al. Synthetic polymeric antibacterial hydrogel for methicillin‐resistant Staphylococcus aureus‐infected wound healing: nanoantimicrobial self‐assembly drug‐ and cytokine‐free strategy. ACS Nano. 2020;14(10):12905‐12917. 32946218 10.1021/acsnano.0c03855

[45] Church D , Elsayed S , Reid O , Winston B , Lindsay R . Burn wound infections. Clin Microbiol Rev. 2006;19(2):403‐434. 16614255 10.1128/CMR.19.2.403-434.2006 PMC1471990

[46] Wei S , Xu P , Yao Z , et al. A composite hydrogel with co‐delivery of antimicrobial peptides and platelet‐rich plasma to enhance healing of infected wounds in diabetes. Acta Biomater. 2021;124:205‐218. 33524559 10.1016/j.actbio.2021.01.046

[47] Rowan MP , Cancio LC , Elster EA , et al. Burn wound healing and treatment: review and advancements. Crit Care. 2015;19:243. 26067660 10.1186/s13054-015-0961-2 PMC4464872

[48] Sun G , Zhang X , Shen YI , et al. Dextran hydrogel scaffolds enhance angiogenic responses and promote complete skin regeneration during burn wound healing. Proc Natl Acad Sci U S A. 2011;108(52):20976‐20981. 22171002 10.1073/pnas.1115973108 PMC3248550

[49] Zhou F , Zhang L , Chen L , et al. Prevascularized mesenchymal stem cell‐sheets increase survival of random skin flaps in a nude mouse model. Am J Transl Res. 2019;11(3):1403‐1416. 30972170 PMC6456548

[50] Sun M , Xie Q , Cai X , et al. Preparation and characterization of epigallocatechin gallate, ascorbic acid, gelatin, chitosan nanoparticles and their beneficial effect on wound healing of diabetic mice. Int J Biol Macromol. 2020;148:777‐784. 31978475 10.1016/j.ijbiomac.2020.01.198

[51] Zhang L , Wang C . Inflammatory response of macrophages in infection. Hepatobiliary Pancreat Dis Int. 2014;13(2):138‐152. 24686541 10.1016/s1499-3872(14)60024-2

[52] Mantovani A , Biswas SK , Galdiero MR , Sica A , Locati M . Macrophage plasticity and polarization in tissue repair and remodelling. J Pathol. 2013;229(2):176‐185. 23096265 10.1002/path.4133

[53] Peng Z , Gao W , Yue B , et al. Promotion of neurological recovery in rat spinal cord injury by mesenchymal stem cells loaded on nerve‐guided collagen scaffold through increasing alternatively activated macrophage polarization. J Tissue Eng Regen Med. 2018;12(3):e1725‐e1736. 27863083 10.1002/term.2358

[54] Kharbikar BN , Chendke GS , Desai TA . Modulating the foreign body response of implants for diabetes treatment. Adv Drug Deliv Rev. 2021;174:87‐113. 33484736 10.1016/j.addr.2021.01.011 PMC8217111

[55] Chen M , Zhang Y , Zhou P , et al. Substrate stiffness modulates bone marrow‐derived macrophage polarization through NF‐κB signaling pathway. Bioactive Mater. 2020;5(4):880‐890. 10.1016/j.bioactmat.2020.05.004 PMC7332470 32637751

[56] Mukherjee T , Balaji KN . The WNT framework in shaping immune cell responses during bacterial infections. Front Immunol. 2019;10:1985. 10.3389/fimmu.2019.01985 PMC6712069 31497020

[57] Abaricia JO , Shah AH , Chaubal M , Hotchkiss KM , Olivares‐Navarrete R . Wnt signaling modulates macrophage polarization and is regulated by biomaterial surface properties. Biomaterials. 2020;243:119920. 32179303 10.1016/j.biomaterials.2020.119920 PMC7191325

[58] Kogut MH , Arsenault RJ . A role for the non‐canonical Wnt‐β‐catenin and TGF‐β signaling pathways in the induction of tolerance during the establishment of a *Salmonella enterica* Serovar Enteritidis persistenst Cecal infection in chickens. Front Vet Sci. 2015;2:33. 10.3389/fvets.2015.00033 PMC4672200 26664962

[59] Cheng C‐W , Yeh J‐C , Fan T‐P , Smith SK , Charnock‐Jones DS . Wnt5a‐mediated non‐canonical Wnt signalling regulates human endothelial cell proliferation and migration. Biochem Biophys Res Commun. 2008;365(2):285‐290. 17986384 10.1016/j.bbrc.2007.10.166

