# Supplementary material for: Bio‐functional hydrogel with antibacterial and anti‐inflammatory dual properties to combat with burn wound infection
Source: Bioeng Transl Med. 2022 Jul 5;8(1):e10373. doi: 10.1002/btm2.10373 (PMC9842067; doi:10.1002/btm2.10373)
Supplement: Supplementary file 1 — Appendix S1 Supporting Information [file BTM2-8-e10373-s001.docx]

**Supporting Information**

**Bio-functional hydrogel with antibacterial and anti-inflammatory dual properties to combat with burn wound infection**

Yahui Xiong ^1, 2, 3^ ^#^, Yingbin Xu ^1, 2, 3^ ^#^, Fei Zhou ^1, 2, 3^, Yanke Hu ^1, 2, 3^, Jingling Zhao ^1, 2, 3^, Zhonghua Liu ^4^, Qiyi Zhai ^5^, Shaohai Qi ^1, 2, 3 *^, Zhaoqiang Zhang ^6^ ^*^, Lei Chen ^1, 2, 3^ ^*^

1. Department of Burns, Laboratory of General Surgery, The First Affiliated Hospital, SunYat-Sen University, Guangzhou, 510080, China

2. Guangdong Provincial Engineering Technology Research Center of Burn and Wound Accurate Diagnosis and Treatment Key Technology and series of products, SunYat-Sen University, Guangzhou, 510080, China

3. Institute of Precision Medicine, The First Affiliated Hospital, SunYat-Sen University, Guangzhou, 510080, China

4. South China Agricultural University, Guangzhou, 510642, China

5. ZhuJiang Hospital, Southern Medical University, 510280, China

6. Department of Oral and Maxillofacial Surgery, Stomatological Hospital, Southern Medical University, No. 366, South of Jiangnan Boulevard, Guangzhou, 510280, China.

^#^ These authors contributed equally to this work.

^*^ Corresponding author: chenlei8@mail.sysu.edu.cn (Lei Chen), 187234415@qq.com (Zhaoqiang Zhang), [qishh@mail.sysu.edu.cn](mailto:qishh@mail.sysu.edu.cn) (Shaohai Qi).

**Table 1**. Primers used for RT-PCR analysis of macrophage phenotype markers

| **Gene** | **Primer** | **Sequence** |
| --- | --- | --- |
| GAPDH | Forward primer | CCTCGTCCCGTAGACAAAATG |
|  | Reverse primer | TGAGGTCAATGAAGGGGTCGT |
| TNF-α | Forward primer | CCCTCACACTCACAAACCACC |
|  | Reverse primer | CTTTGAGATCCATGCCGTTG |
| CD206 | Forward primer | AGTTTGACTTCATCTTCTCCCAGC |
|  | Reverse primer | AGTCCTCCTGTCTGTTGTTCTCAT |


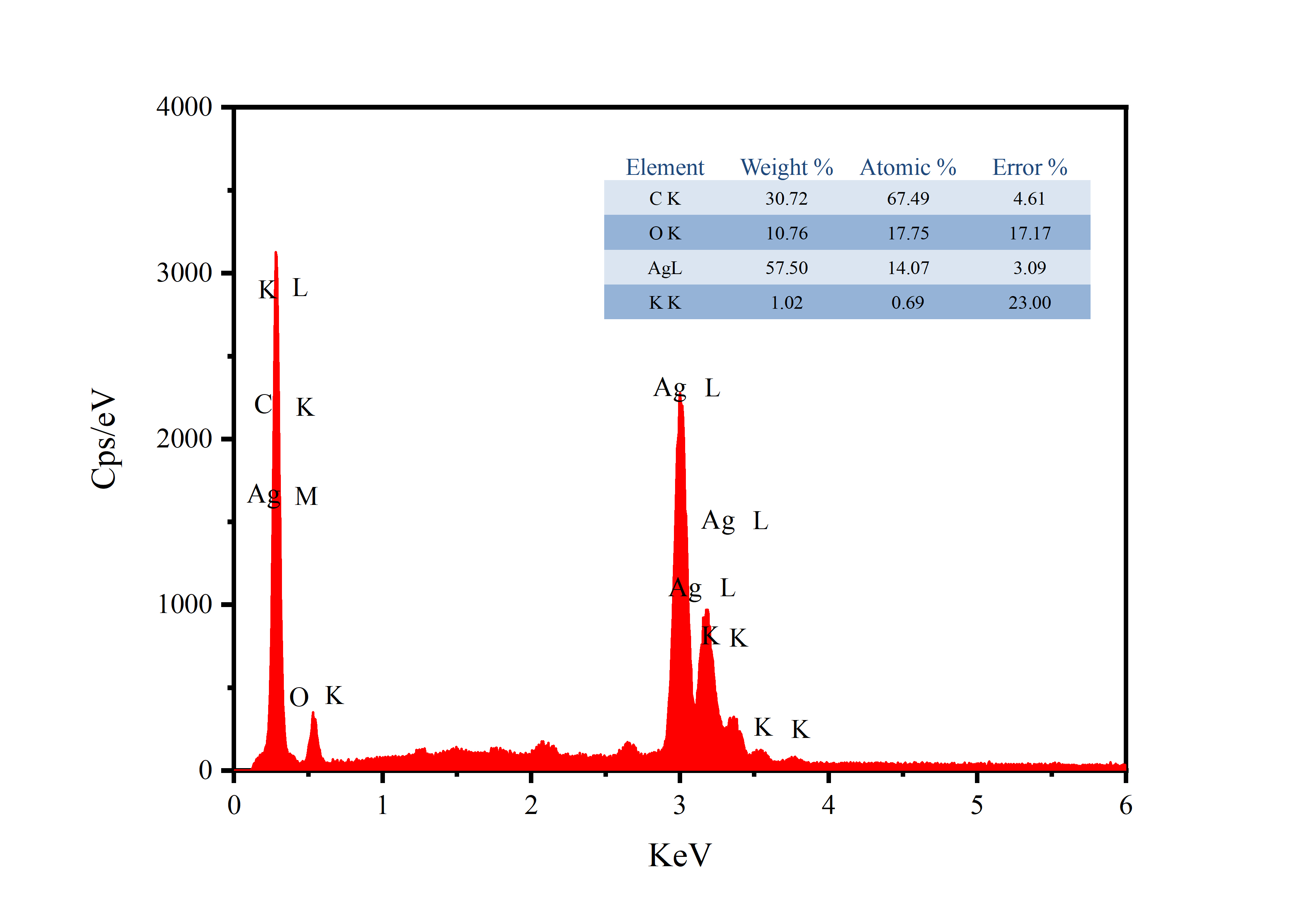


**Figure S1**. The EDS spectrum of Ag@MOF.

**
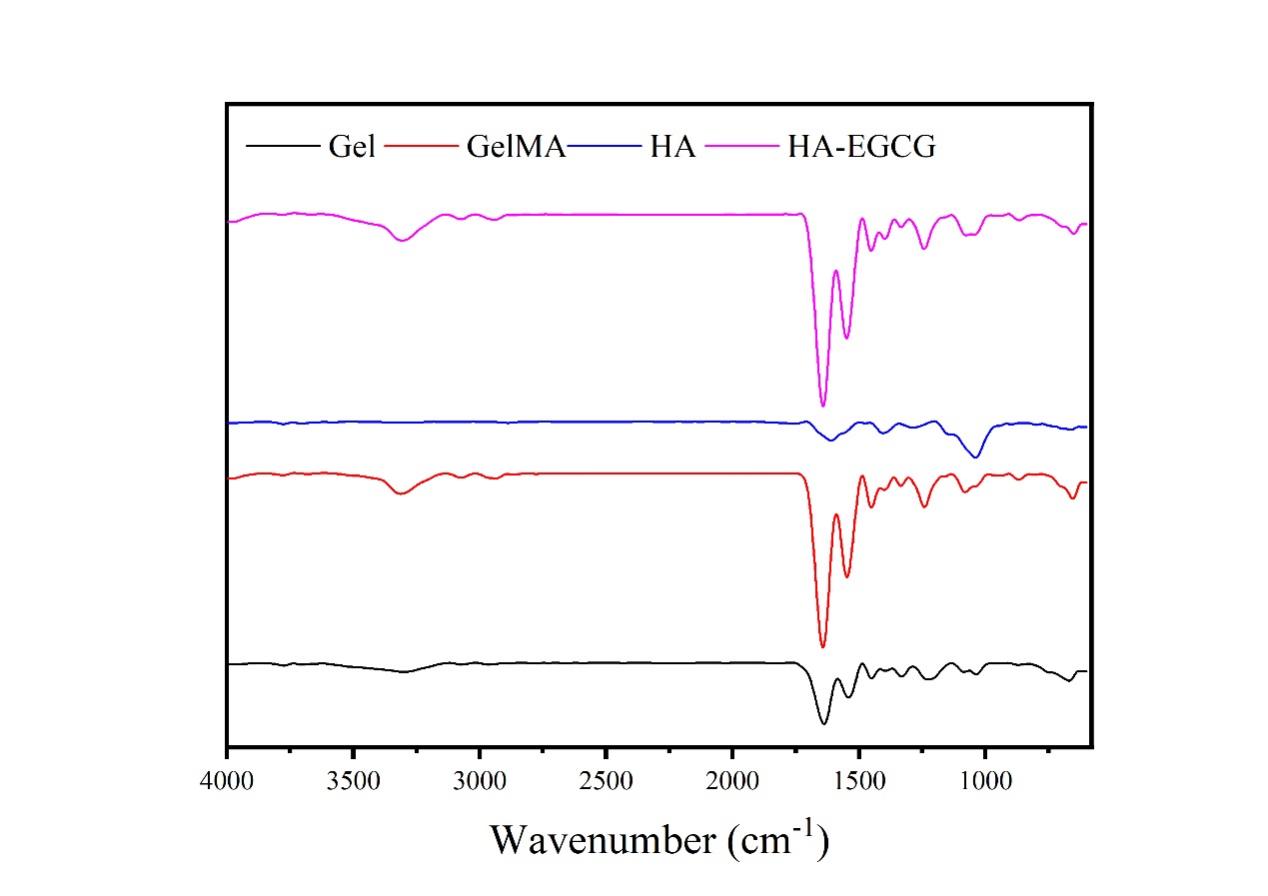
**

**Figure S2.** Fourier-transform infrared spectroscopy (FT-IR) of Gel, GelMA, HA and HA-E.


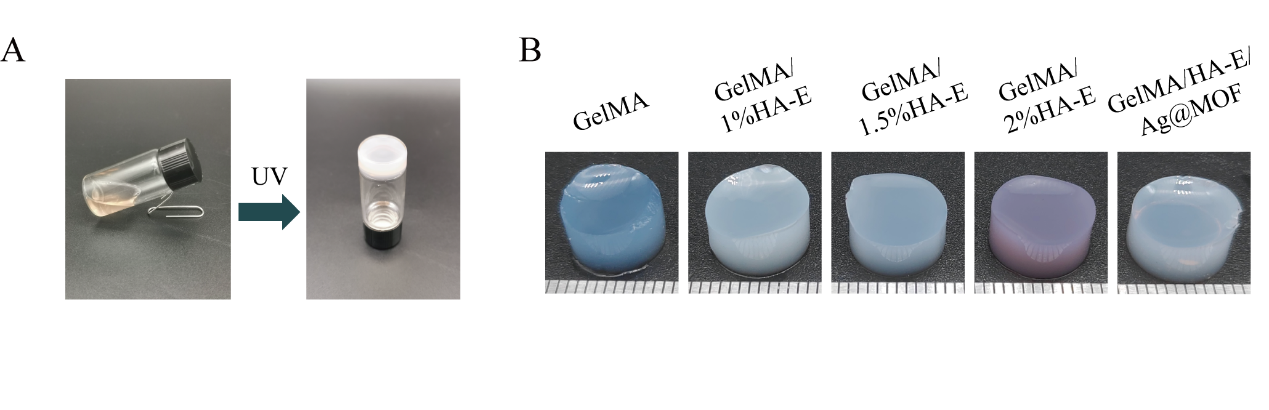


**Figure S3.** (A) Photograph of the GelMA/HA-E hydrogel through a UV crosslinking. (B) The cylindrical GelMA/HA-E hydrogels with different HA-E concentrations.


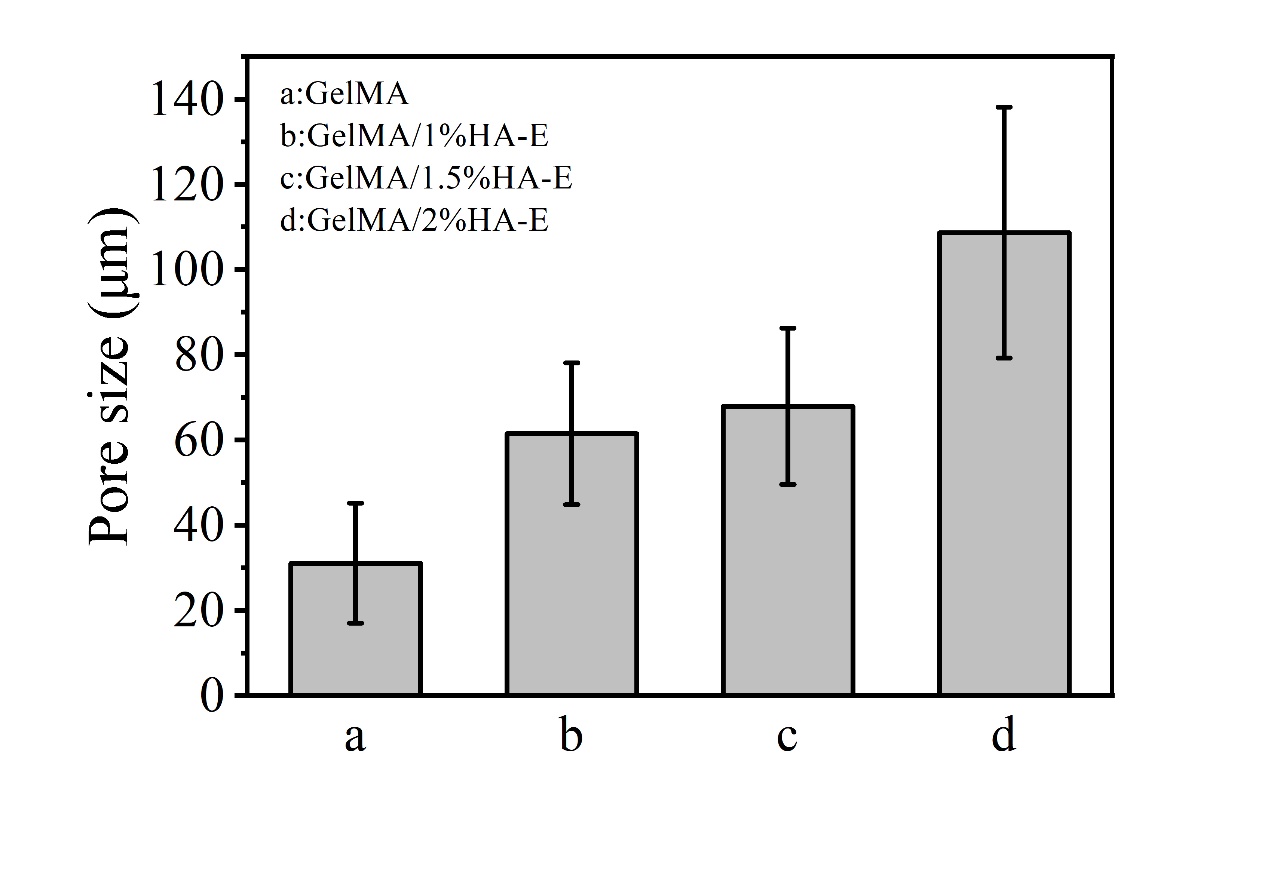


**Figure S4.** The pore size of GelMA/HA-E hydrogels with different HA-E concentration.


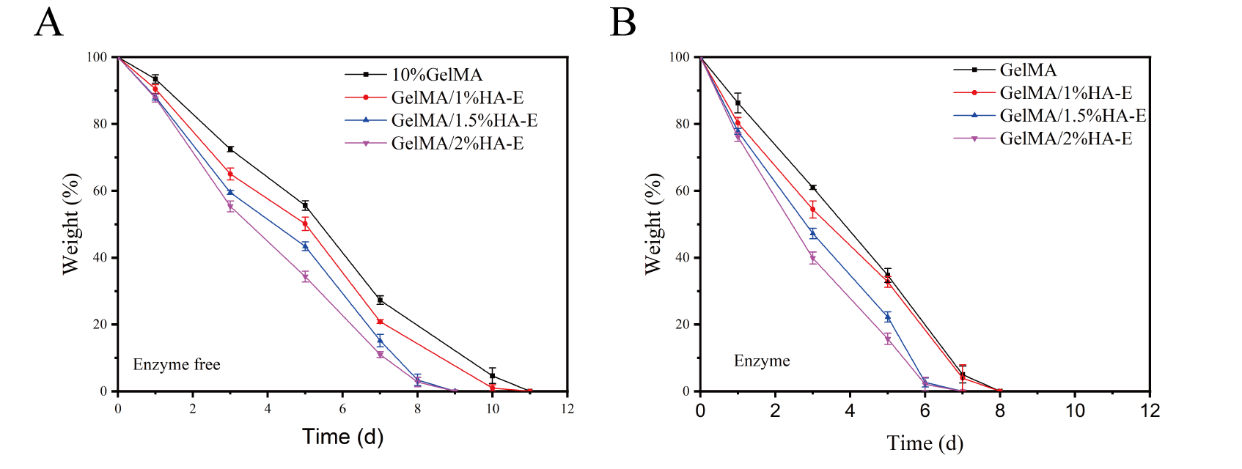


**Figure S5.** (A, B) The weight loss curve of hydrogels in PBS containing either 0 or 100 U/mL of hyaluronidase.

**
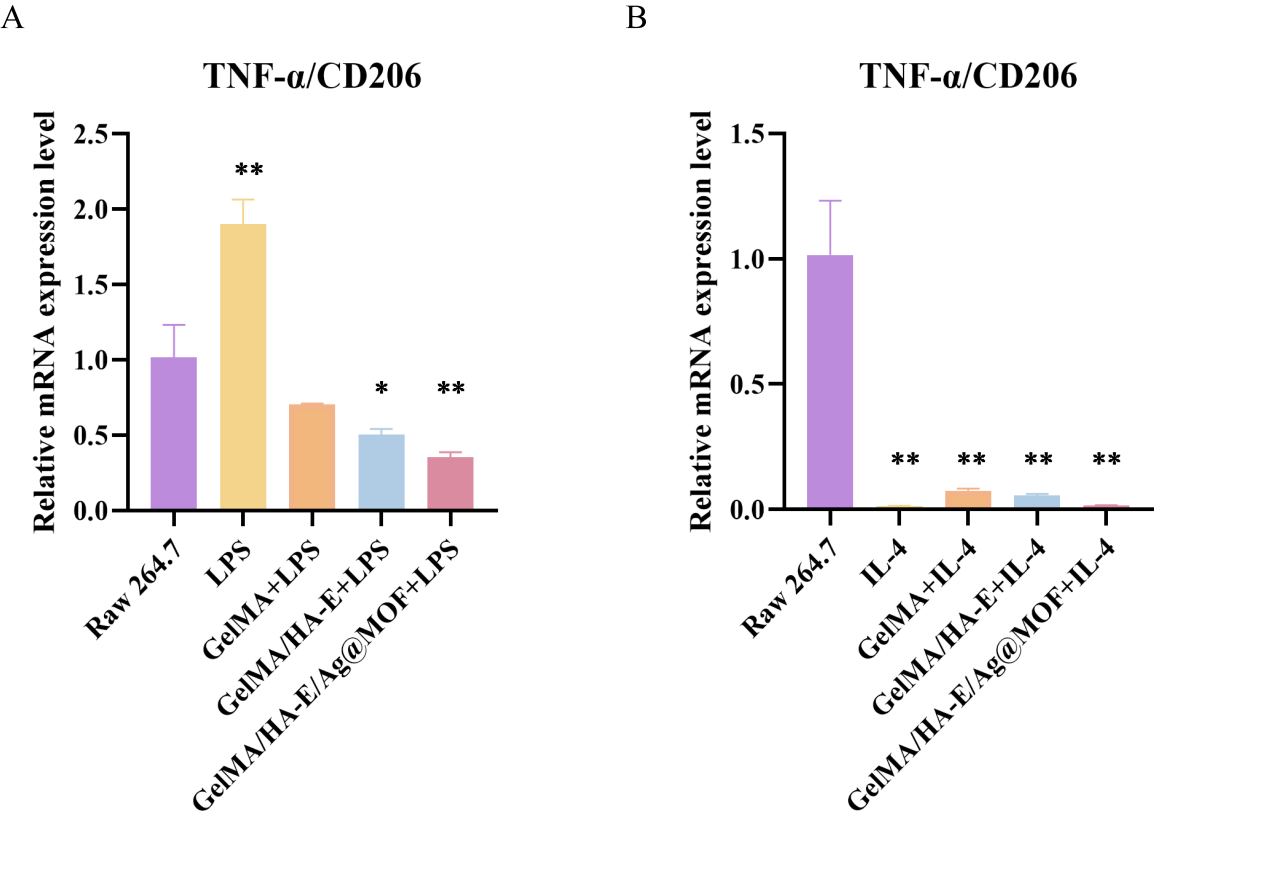
**

**Figure S6.** (A, B) RT-PCR analysis of macrophage-associated marker genes by M1/ M2 *in vitro (*p* < 0.05*, **p*< 0.01*).*

**
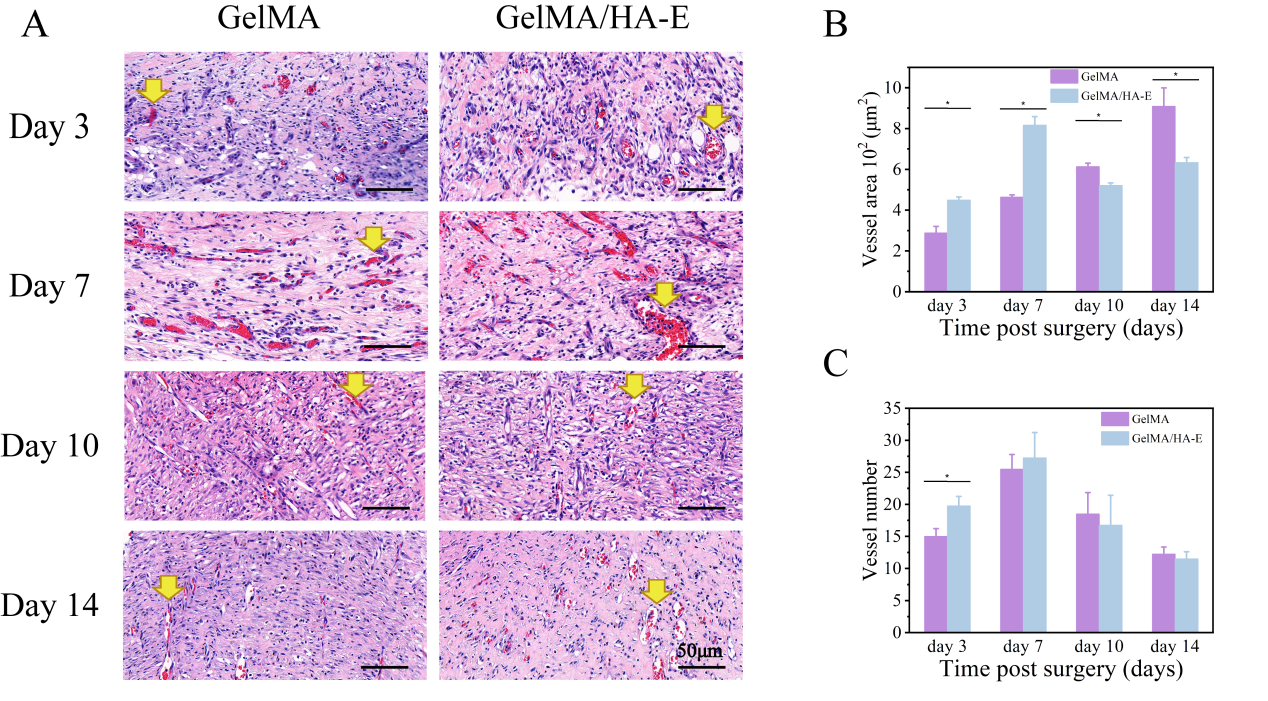
**

**Figure S7.** (A) Representative images of vessels in wound sections of GelMA and GelMA/HA-E hydrogels by HE staining from the 3^rd^ day to the 14^th^ day (indicated by yellow arrows). (B, C) Quantitative analysis of vessel area and vessel number in wounds sections from the 3^rd^ day to the 14^th^ day (**p <* 0.05 ).
